# Low-intensity pulsed ultrasound attenuates atrial remodelling and atrial fibrillation after myocardial infarction: an experimental pre-clinical study

**DOI:** 10.1093/europace/euaf258

**Published:** 2025-10-09

**Authors:** Hongjie Yang, Yugang Hu, Bin Kong, Caijie Shen, Wei Shuai

**Affiliations:** Department of Cardiology, Renmin Hospital of Wuhan University, 238 Jiefang Road, Wuhan, Hubei 430060, P.R. of China; Department of Ultrasound Imaging, Renmin Hospital of Wuhan University, Wuhan , Hubei 430060, P.R. of China; Department of Cardiology, Renmin Hospital of Wuhan University, 238 Jiefang Road, Wuhan, Hubei 430060, P.R. of China; Department of Cardiology, The First Affiliated Hospital of Ningbo University, 59 Liuting Street, Haishu District, Ningbo, Zhejiang Province 315000, China; Department of Cardiology, Renmin Hospital of Wuhan University, 238 Jiefang Road, Wuhan, Hubei 430060, P.R. of China

**Keywords:** Myocardial infarction, Atrial fibrillation, Atrial remodelling, Low-intensity pulsed ultrasound

## Abstract

**Aims:**

Post-infarct atrial remodelling creates a substrate for atrial fibrillation (AF), yet no cardiac-specific, non-invasive therapy targets this process. Low-intensity pulsed ultrasound (LIPUS) limits ventricular remodelling in preclinical models, but its impact on atrial remodelling and AF after myocardial infarction (MI) is unknown.

**Methods and results:**

Myocardial infarction was induced in rats by surgical ligation of the left coronary artery, whereas a separate AF rat model was created by daily tail-vein injections of acetylcholine/calcium chloride (CaCl₂) for 28 days. Low-intensity pulsed ultrasound treatment did not cause significant structural, functional, or electrophysiological changes in the atrial tissue of healthy rats. In MI rats, LIPUS markedly attenuated atrial electrical remodelling, fibrosis, and inflammation, thereby reducing AF susceptibility. Transcriptomic analysis demonstrated a potential role of Adam19/transforming growth factor-β (TGF-β)/Smad2/3 signalling in response to LIPUS treatment, whereas activation of Adam19/TGF-β/Smad2/3 signalling worsened fibrosis and abolished the antiarrhythmic benefit of LIPUS. Similar antifibrotic and antiarrhythmic effects were reproduced in the acetylcholine/CaCl₂ AF model, underscoring LIPUS as a promising non-invasive approach to attenuate AF after MI.

**Conclusion:**

In preclinical post-MI models, LIPUS attenuated atrial structural and electrical remodelling and lowered AF susceptibility, plausibly via modulation of an Adam19/TGF-β/Smad2/3 signalling cascade. These findings are promising but preliminary; priorities include determining whether the atrial effects are direct or secondary to ventricular remodelling and altered haemodynamics, confirming mechanisms across models, defining dose–response and safety and validating efficacy and translational relevance in large-animal studies and early-phase trials before any clinical application.

## Graphical Abstract

**Graphical Abstract:**
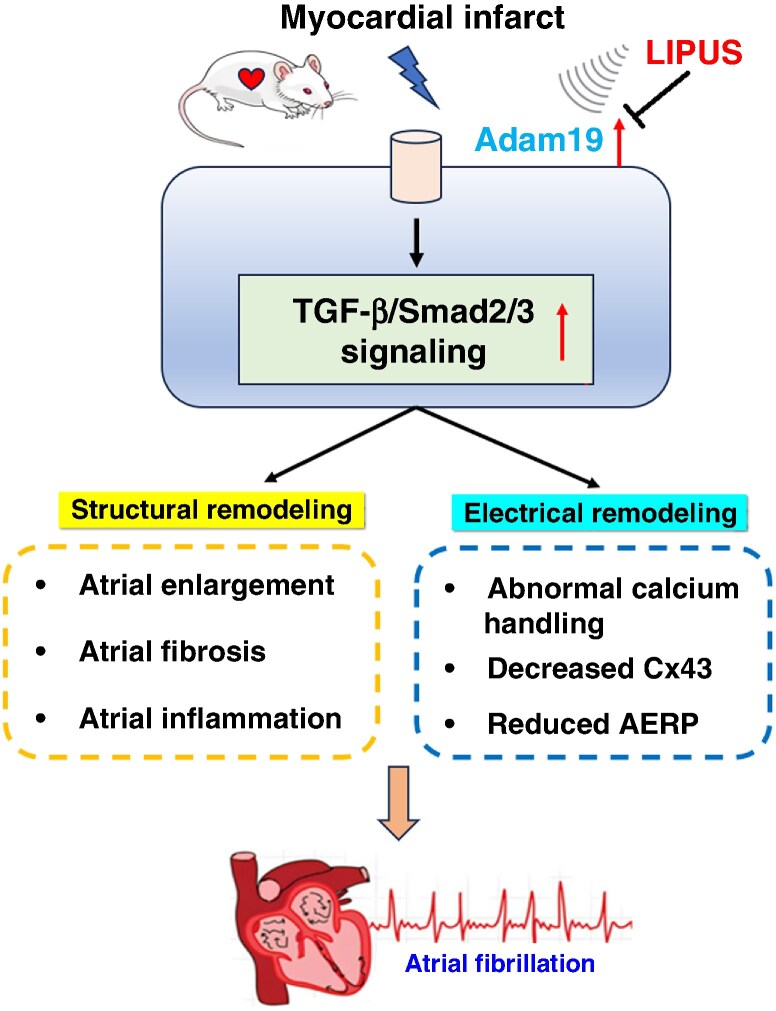

What’s new?

- Low-intensity pulsed ultrasound treatment reduced susceptibility to atrial fibrillation following myocardial infarction.
- Low-intensity pulsed ultrasound left cardiac structure, function, and atrial electrophysiology unaltered in sham rats, supporting a preliminary safety margin for translation.
- Low-intensity pulsed ultrasound treatment significantly attenuated myocardial infarction-induced left atrial electrical and structural remodelling.
- Low-intensity pulsed ultrasound modulated an Adam19/transforming growth factor-β/Smad2/3 signalling cascade in atrial tissue, consistent with reduced profibrotic signalling.
- Low-intensity pulsed ultrasound likewise curbed fibrosis and arrhythmia in an acetylcholine/calcium chloride atrial fibrillation (AF) model, suggesting its broad, non-invasive potential for attenuate post-myocardial infarction AF.

## Introduction

Atrial fibrillation (AF), which has an incidence of 6–21% in myocardial infarction (MI) patients, is the most common supraventricular arrhythmia, and acute MI increases the risk of arrhythmia.^1,2^ Stroke, heart failure, and death are more likely to occur in individuals with AF complicating MI than in MI patients without AF.^3,4^ The particular mechanisms driving AF post-MI, however, were largely unclear until recently. Atrial overstretching, inflammation, fibrosis, and hormonal and neurological system activation were all linked to AF post-MI, according to earlier investigations.^5,6^ The structural and electrical remodelling of the atrium is a need for the onset of AF. Investigating therapeutic strategies that can impede this remodelling may provide a viable approach to preventing AF following MI.

Recent studies have explored the efficacy of low-intensity pulsed ultrasound (LIPUS) therapy in modulating structural changes in the left ventricle, such as angiogenesis, hypertrophy, fibrosis, and inflammation, within the context of various cardiovascular diseases.^7–9^ Additionally, LIPUS treatment has been proposed to mitigate electrical remodelling in cases of heart failure with preserved left ventricular ejection fraction (HFpEF) by modulating calcium handling in the myocardium.^8^ However, the potential effects of LIPUS treatment on atrial remodelling and atrial arrhythmia have not been explored. In this study, we aimed to assess whether LIPUS treatment could attenuate AF and atrial remodelling induced by MI and to elucidate the underlying mechanisms involved.

## Methods

Methods can be found in the *Supplementary material*.

## Results

### Low-intensity pulsed ultrasound treatment reduced atrial fibrillation susceptibility

**Figure 1:**
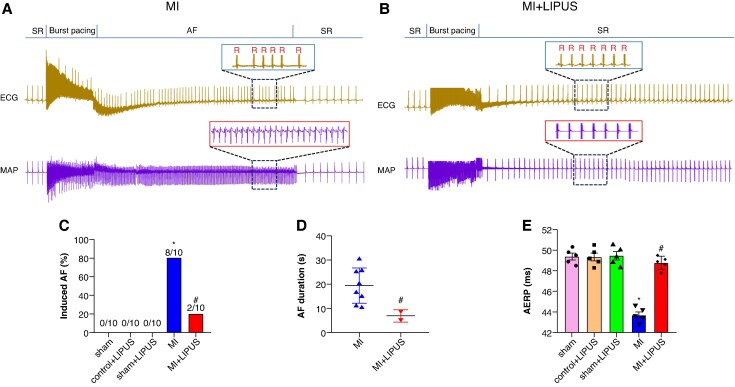
LIPUS treatment alleviated MI-induced AF susceptibility. (*A–B*) Representative examples of AF induced by burst stimulating in the MI group and the MI + LIPUS group. (*C*) Ratio of burst-induced AF and (*D*) duration of AF (*n* = 10 per group). (*E*) Quantification of AERP (*n* = 5 per group). *P < 0.05 vs. sham group. #*P* < 0.05 vs. MI group. Statistics: AF incidence by two-tailed Fisher’s exact test; all other panels by one-way analysis of variance (ANOVA) with Tukey’s multiple-comparisons test. AERP, atrial effective refractory period; AF, atrial fibrillation; LIPUS, low-intensity pulsed ultrasound; MI, myocardial infarction.

We first examined the effect of LIPUS treatment on AF susceptibility in post-MI rats. *Figure 1A* and *B* exhibited the representative images of burst-induced AF in MI group and MI + LIPUS group. The results showed that the AF induction rate in the MI group was significantly increased compared to the sham group (80% vs. 0%, *Figure 1C*), LIPUS has no effect on healthy rats, LIPUS treatment significantly reduced AF susceptibility compared to MI group (20% vs. 80%, *Figure 1C*). Moreover, LIPUS treatment significantly reduced the duration of AF compared to the MI group (7.05 ± 2.17 s vs. 20.16 ± 8.47 s; *Figure 1D*). In addition, atrial abnormal electrical remodelling underlies the pathologic basis of AF occurrence. We also checked the effective refractory period (ERP) in rats, the results suggested that the atrial effective refractory period (AERP) was shortened in the MI group, and LIPUS treatment modestly but significantly increased AERP compared to the MI group (*Figure 1E*). Collectively, these data suggested that LIPUS treatment could reduce MI-induced AF susceptibility.

Meanwhile, to further clarify the potential effect of LIPUS on AF inducibility, another AF rat model [acetylcholine/calcium chloride (ACh/CaCl₂)-induced AF rat model] was produced. Low-intensity pulsed ultrasound treatment significantly reduced AF susceptibility compared to AF group (10% vs. 80%, Supplementary material online, *Figure S1A* and *B*). Low-intensity pulsed ultrasound treatment significantly reduced the duration of AF compared to the AF group (11.6 s vs. 21.91 ± 5.61 s; Supplementary material online, *Figure S1C*). We also checked the ERP in rats, the results suggested that the AERP was shortened in the AF group, and LIPUS treatment significantly increased AERP compared to the AF group (see Supplementary material online, *Figure S1D*).

### Low-intensity pulsed ultrasound treatment attenuated atrial enlargement

**Figure 2:**
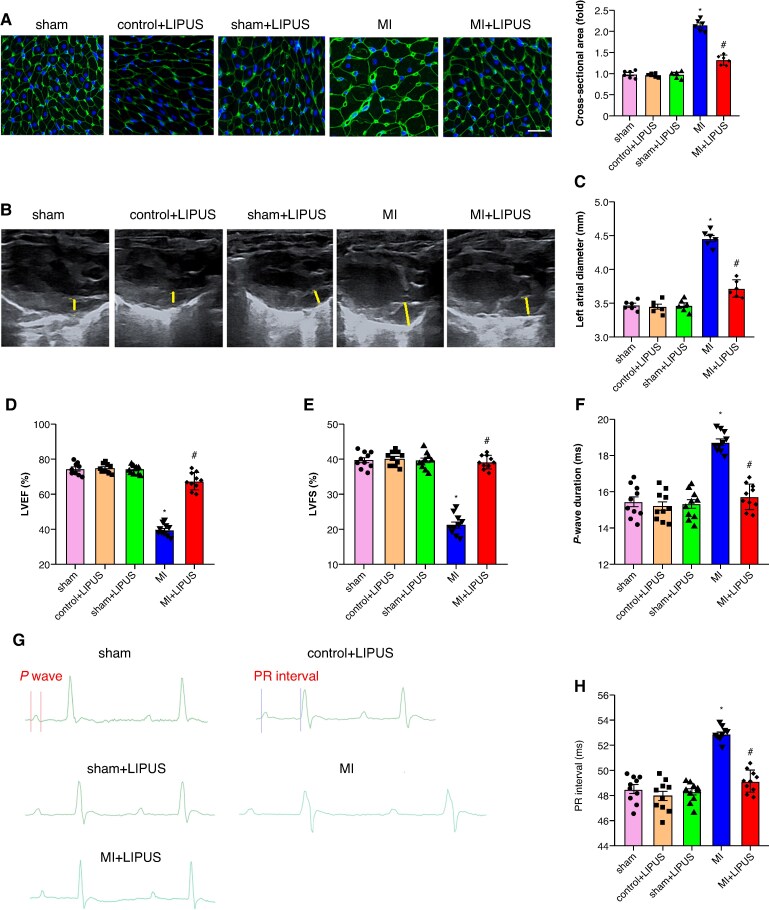
LIPUS treatment attenuated MI-induced atrial enlargement. (*A*) Representative WGA staining in atrial and quantitative results cross-sectional area (*n* = 6 per group). (*B–C*) Representative echocardiography images of LAD and quantification of LAD (*n* = 6 per group). (*D*) LVEF (*n* = 10 per group). (*E*) LVFS (*n* = 10 per group). (*F*) Representative ECG images. (*G*) Quantification of *P* wave duration and (*H*) duration of PR interval (*n* = 10 per group). *P < 0.05 vs. sham group. #*P* < 0.05 vs. MI group. Statistics: one-way ANOVA with Tukey’s multiple-comparisons test. LAD, left atrial diameter; LVEF, left ventricular ejection fraction (*n* = 10 per group); LVFS, left ventricular fractional shortening (*n* = 10 per group); MI, myocardial infarction; WGA, wheat germ agglutinin.

Echocardiography, histology, and electrocardiography were performed 4 weeks following MI to evaluate the effect of LIPUS treatment on MI-induced atrial enlargement, after which the rats were sacrificed. Wheat germ agglutinin (WGA) staining revealed a significant increase in cross-sectional area in MI atrium compared to sham atrium, LIPUS treatment notably decreased cross-sectional area compared to MI atrium (*Figure 2A*). Echocardiography parameter left atrial diameter (LAD), left ventricular ejection fraction (LVEF), and fractional shortening (LVFS) were also assessed, and the results demonstrated that increased LAD and decreased LVEF and LVFS were found in MI rats, and LIPUS treatment markedly decreased LAD and increased LVEF and LVFS compared to MI rats (*Figure 2B–D*). Meanwhile, abnormal electrocardiogram (ECG) parameters contribute to AF vulnerability; we then tested the effect of LIPUS treatment on ECG parameters. As shown in *Figure 2F–H*, the *P* wave and PR duration were markedly increased in the MI group, and LIPUS treatment notably decreased the *P* wave and PR duration (*Figure 2F–H*). Taken together, these results indicated that LIPUS treatment can attenuate MI-induced atrial enlargement.

Another AF rat model also performed to verify the effect of LIPUS treatment on atrial enlargement. Wheat germ agglutinin staining revealed a significant increase of cross-sectional area in the AF atrium compared to the vehicle atrium, and LIPUS treatment notably decreased the cross-sectional area compared to the AF atrium (see Supplementary material online, *Figure S2A*). Echocardiography parameter LAD was also assessed, and the results demonstrated that increased LAD was found in AF rats, and LIPUS treatment markedly decreased LAD compared to AF rats (see Supplementary material online, *Figure S2B*). Moreover, the *P* wave and PR duration were markedly increased in the AF group, and LIPUS treatment notably decreased the *P* wave and PR duration (see Supplementary material online, *Figure S2C* and *D*).

### Low-intensity pulsed ultrasound treatment mitigated atrial structural remodelling

**Figure 3:**
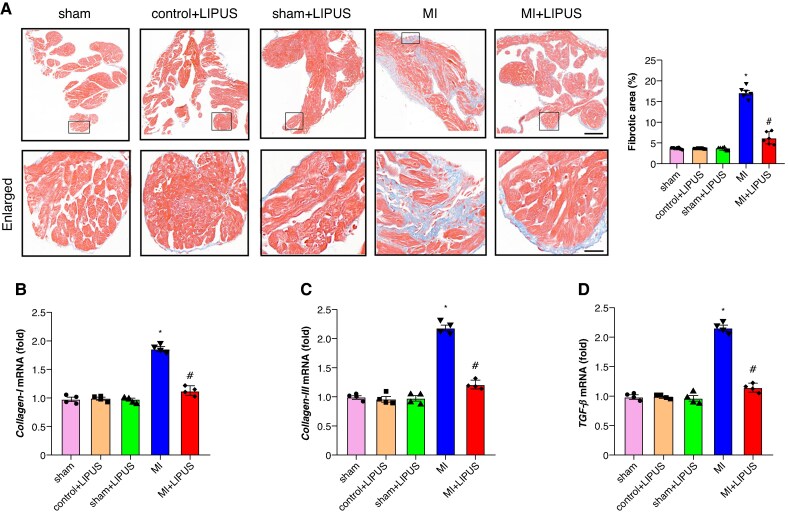
LIPUS treatment ameliorates atrial fibrosis induced by MI. (*A*) Representative images of Masson-stained heart sections and quantification of percentage of the fibrotic area (*n* = 6 per group). (*B–D*) Quantification of mRNA levels of *collagen-I*, *collagen-III*, and *TGF-β* (*n* = 4 per group). *P < 0.05 vs. sham group. #*P* < 0.05 vs. MI group. Statistics: one-way ANOVA with Tukey’s multiple-comparisons test. LIPUS, low-intensity pulsed ultrasound; MI, myocardial infarction; TGF-β, transforming growth factor-β.

**Figure 4:**
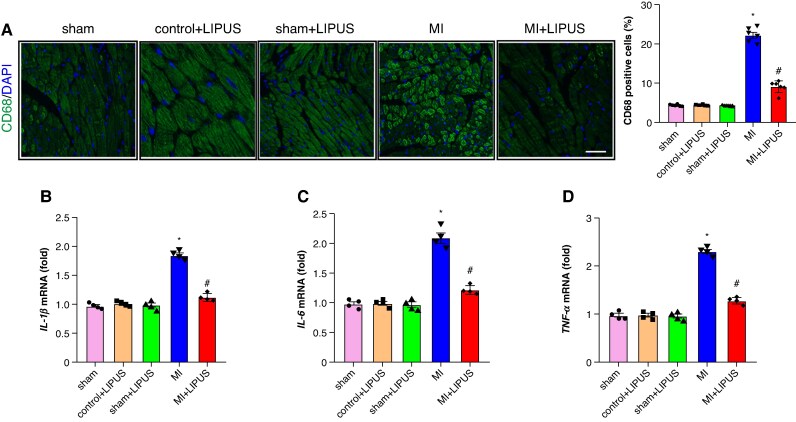
LIPUS treatment ameliorates atrial inflammation induced by MI. (*A*) Representative CD68 immunofluorescence and quantitative results of CD68-positive cells (*n* = 6 per group). (*B–D*) Quantification of mRNA levels of *IL-1β*, *IL-6*, and *TNF-α* (*n* = 4 per group). *P < 0.05 vs. sham group. #*P* < 0.05 vs. MI group. Statistics: one-way ANOVA with Tukey’s multiple-comparisons test. IL-1β, interleukin-1β; IL-6, interleukin-6; LIPUS, low-intensity pulsed ultrasound; MI, myocardial infarction; TNF-α, tumour necrosis factor-α.

Histological analysis was performed to check the effect of LIPUS on atrial fibrosis and inflammation induced by MI. The results demonstrated enhanced atrial fibrosis and inflammation in the MI atrium compared to the sham atrium, as illustrated in *Figures 3A* and *4A*. Low-intensity pulsed ultrasound treatment significantly mitigated the MI-induced atrial fibrosis and inflammation, evidenced by a reduction in the fibrotic area and the number of CD68-positive cells (*Figures 3A* and *4A*). Furthermore, reverse transcription-polymerase chain reaction (RT-qPCR) analysis was employed to further assess the impact of LIPUS treatment on atrial structural remodelling induced by MI. As depicted in *Figure 3B–D*, there was a significant upregulation of collagen-I, collagen-III, and transforming growth factor-β (TGF-β) mRNA in the MI atrium, which was notably decreased by LIPUS treatment compared to the MI group. Additionally, elevated levels of IL-1β, IL-6, and TNF-α mRNA observed in the MI atrium were significantly reduced following LIPUS treatment compared to the MI group, as shown in *Figure 4B–D*. Overall, these findings demonstrate that LIPUS treatment effectively attenuates MI-induced atrial structural remodelling, including reductions in atrial fibrosis and inflammation.

We also explored the effect of LIPUS treatment on ACh/CaCl₂-induced atrial fibrosis and inflammation. Masson staining and RT-qPCR analysis indicated that LIPUS treatment significantly attenuated ACh/CaCl_2_-induced cardiac fibrosis (see Supplementary material online, *Figure S3A*–*D*). Meanwhile, CD68 immunofluorescence and RT-qPCR analysis demonstrated that LIPUS treatment significantly alleviated ACh/CaCl_2_-induced cardiac inflammatory response (see Supplementary material online, *Figure S4A*–*D*).

Furthermore, the results of HL-1 experiments are consistent with those of animal experiments, and LIPUS treatment significantly attenuated oxygen-glucose deprivation (OGD)-induced profibrotic gene expression, as reflected by decreasing collagen-I, collagen-III, and TGF-β mRNA (see Supplementary material online, *Figure S5A*–*C*). Low-intensity pulsed ultrasound treatment significantly attenuated OGD-induced cardiomyocyte inflammatory response, as reflected by decreasing IL-1β, IL-6, and TNF-α mRNA (see Supplementary material online, *Figure S5D*–*F*).

### Low-intensity pulsed ultrasound treatment alleviated abnormalities in atrial calcium handling protein induced by myocardial infarction

**Figure 5:**
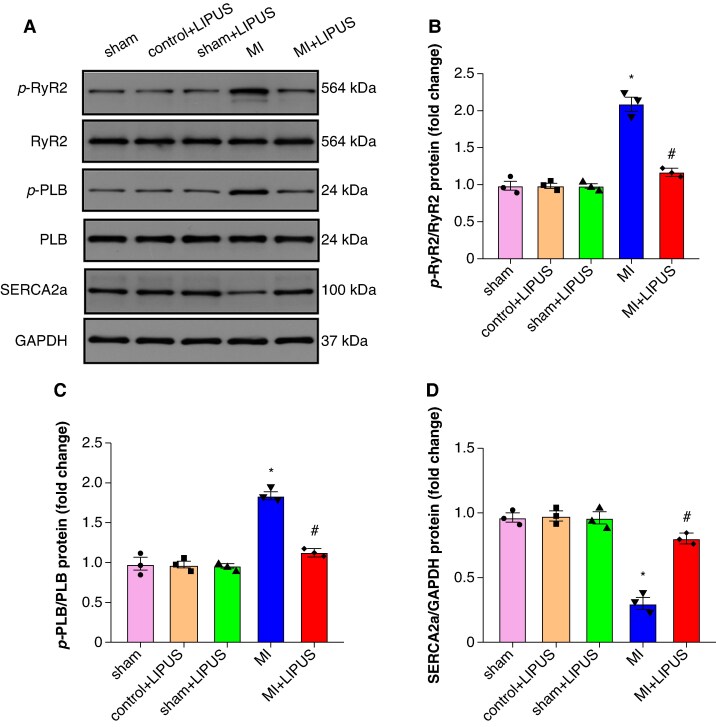
LIPUS treatment attenuated MI-induced atrial electrical remodelling. (*A*) Representative western blot and (*B–D*) statistical results of p-RyR2/RyR2, p-PLB/PLB, and SERCA2a protein expression (*n* = 3 per group). *P < 0.05 vs. sham group. #*P* < 0.05 vs. MI group. Statistics: one-way ANOVA with Tukey’s multiple-comparisons test. LIPUS, low-intensity pulsed ultrasound; MI, myocardial infarction; PLB, phospholamban; RyR2, ryanodine receptor 2; SERCA2a, sarco-/endoplasmic reticulum Ca2+-ATPase 2a.

We further examined the protein expression of sarco-/endoplasmic reticulum Ca2+-ATPase 2a (SERCA2a), phospholamban (PLB), and ryanodine receptor 2 (RyR2) in the atrium. In the MI atrium compared to the sham group, there was an elevation in phosphorylated ryanodine receptor 2 (p-RyR2) and phosphorylated phospholamban (p-PLB) levels, along with a down-regulation of SERCA2a protein expression. Low-intensity pulsed ultrasound treatment significantly decreased the levels of p-RyR2 and p-PLB and increased SERCA2a protein expression (*Figure 5A–D*). Altogether, these data demonstrated that LIPUS treatment significantly alleviated MI-induced abnormalities in atrial calcium handling protein. Additionally, the results of HL-1 experiments are consistent with those of animal experiments, and LIPUS treatment significantly decreased the levels of p-RyR2 and p-PLB and increased SERCA2a protein expression induced by OGD (see Supplementary material online, *Figure S6A*–*D*).

### Low-intensity pulsed ultrasound treatment increased connexin 43 expression

**Figure 6:**
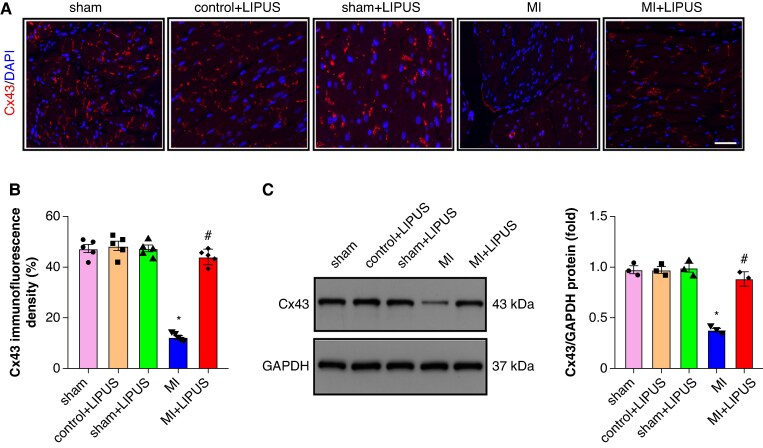
LIPUS treatment regulated MI-induced gap junction remodelling. (*A–B*) Typical examples and quantitative results of Cx43 immunofluorescence staining (*n* = 5 per group). (*C*) Representative western blot and statistical results of Cx43 protein expression (*n* = 3 per group). *P < 0.05 vs. sham group. #*P* < 0.05 vs. MI group. Statistics: one-way ANOVA with Tukey’s multiple-comparisons test. Cx43, connexin 43; LIPUS, low-intensity pulsed ultrasound; MI, myocardial infarction.

Connexin 43 (Cx43) is the predominant gap junction protein in the atrium, associated with atrial conduction. As shown in *Figure 6A* and *B*, significantly reduced Cx43 immunofluorescence intensity was seen in the MI atrium compared to the sham group, and LIPUS treatment could significantly increase Cx43 immunofluorescence intensity compared to the MI group. Western blot analysis revealed a reduction in Cx43 protein expression in the MI group compared to the sham group, and LIPUS treatment significantly increased the Cx43 protein expression compared to the MI group (*Figure 6C*).

In addition, we evaluated the effect of LIPUS treatment on Cx43 expression. Connexin 43 immunofluorescence revealed that LIPUS treatment significantly increased the Cx43 immunofluorescence intensity compared to the AF group (see Supplementary material online, *Figure S7*). However, ACh/CaCl_2_ did not have an effect on cardiac function, ventricular hypertrophy, fibrosis, and ventricular arrhythmias inducibility, and LIPUS has no significant effect on ACh/CaCl_2_-induced ventricular phenotype (see Supplementary material online, *Figure S8A*–*F)*.

### Adam19 is a potential target of low-intensity pulsed ultrasound treatment

**Figure 7:**
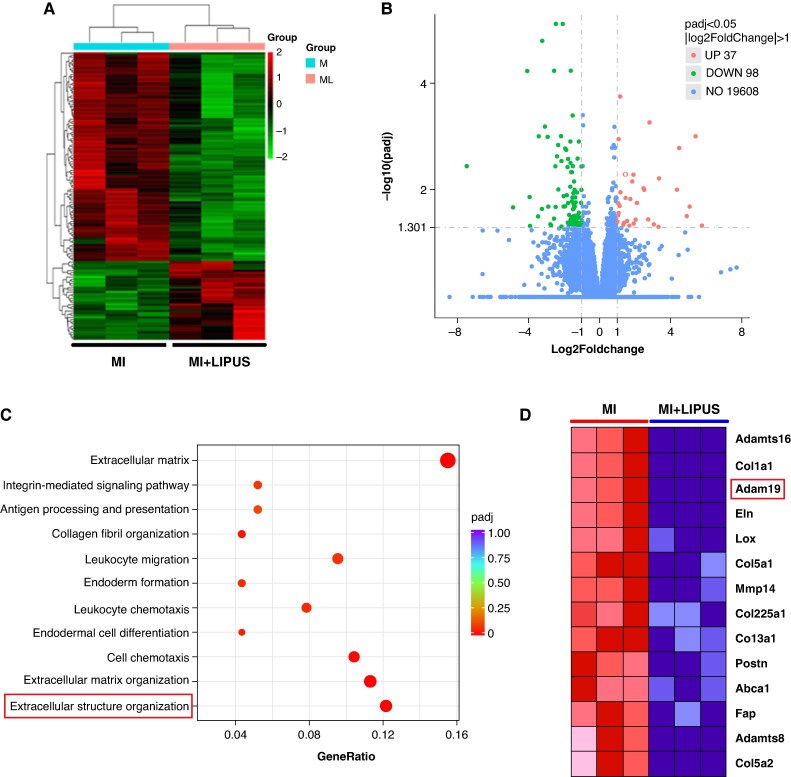
RNA-seq analysis. (*A*) Heatmap of RNA-seq expression levels of genes in MI vs. MI + LIPUS atrium (*n* = 3 per group). (*B*) Volcano plot showed the DEGs in the atrium between MI and MI + LIPUS rats. (*C*) GO enrichment analysis. (*D*) DEGs associated with extracellular structure organization. Statistics: differential expression and enrichment analyses were performed as detailed in the Methods section with multiple-testing correction. DEGs, differentially expressed genes; GO, Gene Ontology; LIPUS, low-intensity pulsed ultrasound; MI, myocardial infarction; RNA-seq = RNA sequencing.

To explore the potential mechanisms underlying the protective effects of LIPUS treatment, transcriptome profiles of atrial tissue in the MI group or MI + LIPUS group to determine which genes are differentially expressed in the atrium between the MI group and MI + LIPUS group using RNA sequencing (RNA-seq) analysis. The heat map indicated the differential expression genes (DEGs; *Figure 7A*). After 4-week LIPUS treatment, 37 genes were significantly up-regulated, while 98 genes were down-regulated compared to the MI group (*Figure 7B*). Gene Ontology (GO) enrichment analysis showed significant biological processes, including extracellular structure organization, extracellular matrix organization, cell chemotaxis, endodermal cell differentiation, and leucocyte chemotaxis (*Figure 7C*). In the two significant biological processes, including extracellular structure organization and extracellular matrix organization, Adam19 (a disintegrin and metalloproteinase 19) was the one most DEGs related to extracellular structure organization and extracellular matrix organization between the MI group and the MI + LIPUS group, which aroused our great interest (*Figure 7D*).

**Figure 8:**
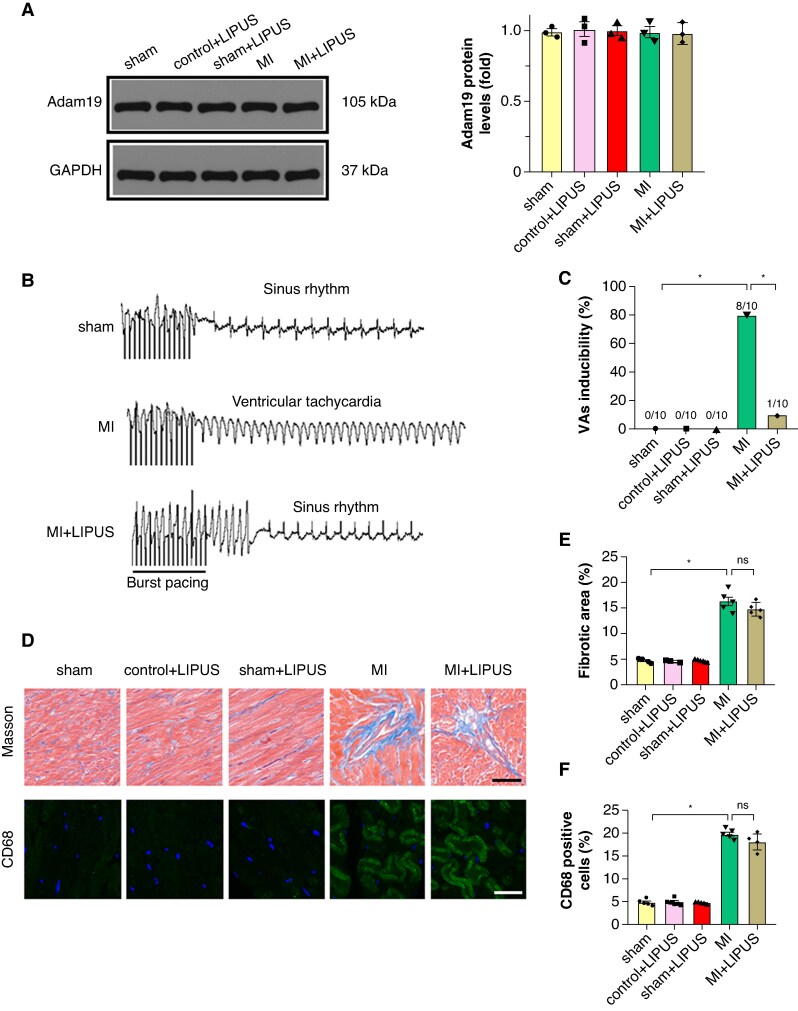
The effect of LIPUS treatment on MI-induced cardiac remodelling. (*A*) Representative western blot and statistical results of ventricular Adam19 protein expression (*n* = 3). (*B–C*) Representative examples of VAs induced by burst-stimulating and statistical analysis (*n* = 10 per group). **(***D–E***)** Representative images of Masson-stained heart sections and quantification of percentage of the fibrotic area (*n* = 5 per group). (*F*) Representative CD68 immunofluorescence and quantitative results of CD68-positive cells (*n* = 5 per group). Statistics: one-way ANOVA with Tukey’s multiple-comparisons test. Adam19, a disintegrin and metalloproteinase 19; LIPUS, low-intensity pulsed ultrasound; MI, myocardial infarction; VAs, ventricular arrhythmias.

**Figure 9:**
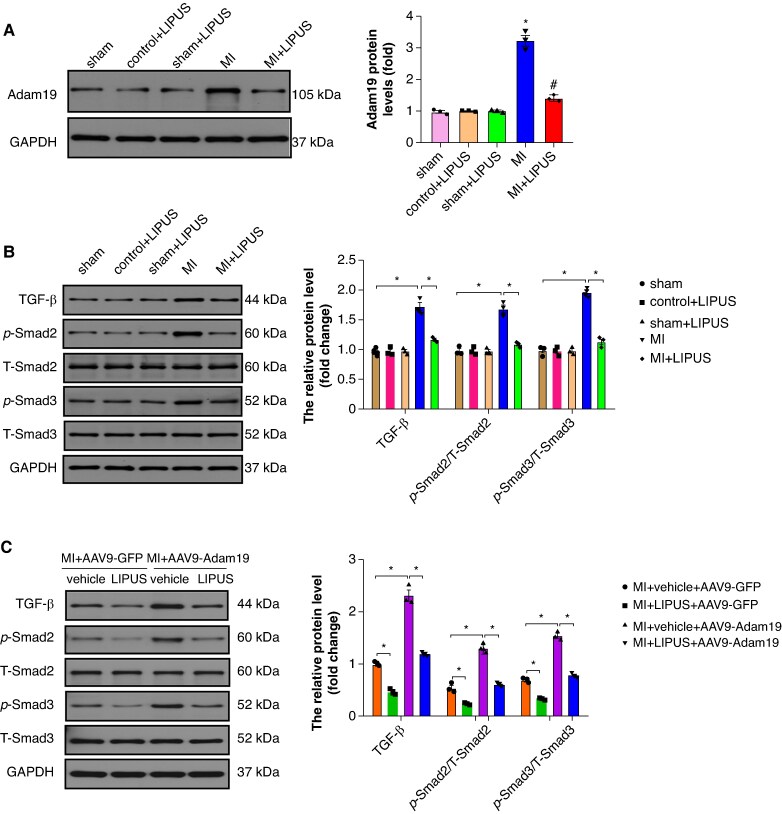
LIPUS treatment down-regulated Adam19 and inactivated TGF-β/Smad2/3 signalling pathway. (*A*) Representative western blot and statistical results of Adam19 protein expression (*n* = 3 per group). *P < 0.05 vs. sham group. #*P* < 0.05 vs. MI group. (*B*) Representative western blot and statistical results of TGF-β, p-Smad2, T-Smad2, p-Smad3, and T-Smad3 protein expression (*n* = 3 per group). (*C*) Representative western blot and statistical results of TGF-β, p-Smad2, T-Smad2, p-Smad3, and T-Smad3 protein expression after AAV9 injection (*n* = 3 per group). *P < 0.05. Statistics: one-way ANOVA with Tukey’s multiple-comparisons test. AAV9, adeno-associated virus 9; Adam19, a disintegrin and metalloproteinase 19; LIPUS, low-intensity pulsed ultrasound; MI, myocardial infarction; TGF-β, transforming growth factor-β.

To assess changes in Adam19 within the atrium, we performed western blot analysis. Adam19 protein levels were significantly increased in the atrium after MI compared with sham, whereas LIPUS significantly reduced atrial Adam19 relative to MI (*Figure 9A*). In contrast, ventricular Adam19 protein levels remained unchanged across groups (*Figure 8A*). In the MI model, LIPUS preserved both atrial and ventricular functions. Meanwhile, we observed that LIPUS improved left ventricular systolic function and reduced susceptibility to ventricular arrhythmias (*Figure 8B* and *C*), but did not significantly affect ventricular fibrosis and inflammation (*Figure 8D–F*). These findings argue against a dominant ventricular Adam19-mediated mechanism, but do not exclude ventricular, Adam19-independent pathways contributing to the atrial phenotype.

### Adam19/TGF-β/Smad2/3 signalling cascades contributes to the protective role of low-intensity pulsed ultrasound

Adam19 is a stress-responsive metalloproteinase whose ectodomain-shedding activity activates latent TGF-β, thereby driving the fibroblast-to-myofibroblast transition and promoting atrial fibrosis after myocardial injury.^10–12^ We next evaluated whether the Adam19/TGF-β/Sma and Mad-related protein (Smad)2/3 pathway was activated in the atrium. Adeno-associated virus 9 (AAV9) was used to overexpress Adam19 in the atrium. Western blot analysis revealed that LIPUS treatment significantly inhibited the TGF-β/Smad2/3 signalling pathway compared to the MI group, while Adam19 overexpression significantly further activated the TGF-β/Smad2/3 signalling pathway compared to the MI group and abolished the inhibition effect of LIPUS treatment on the TGF-β/Smad2/3 signalling pathway (*Figure 9B* and *C*), which manifested that Adam19 could activate the TGF-β/Smad2/3 signalling pathway.

To further verify the potential effect of LIPUS on Adam19/TGF-β/Smad2/3 signalling cascades, we added a cell-autonomous HL-1 atrial myocyte OGD model with six groups: PBS, OGD, OGD + LIPUS, OGD + LIPUS + Ad-Adam19, OGD + LIPUS + Ad-Adam19 + BB-94 (a specific inhibitor of matrix metalloproteinases), and OGD + LIPUS + Ad-Adam19 + SB431542 (a specific inhibitor of TGF-β). We quantified TGF-β/Smad2/3 signalling by western blot (see Supplementary material online, *Figure S9*), and the results indicated that LIPUS attenuated OGD-induced profibrotic signalling, Ad-Adam19 blunted these benefits. Both BB-94 and SB431542 restored the LIPUS effect, consistent with Adam19 driving TGF-β/Smad2/3 signalling. These results suggest that LIPUS may exert protective effects by regulating the Adam19/TGF-β/Smad2/3 signalling pathway.

### Activation of Adam19/TGF-β/Smad2/3 signalling counteracted the protective effect of low-intensity pulsed ultrasound treatment on myocardial infarction-induced atrial electrical remodelling

**Figure 10:**
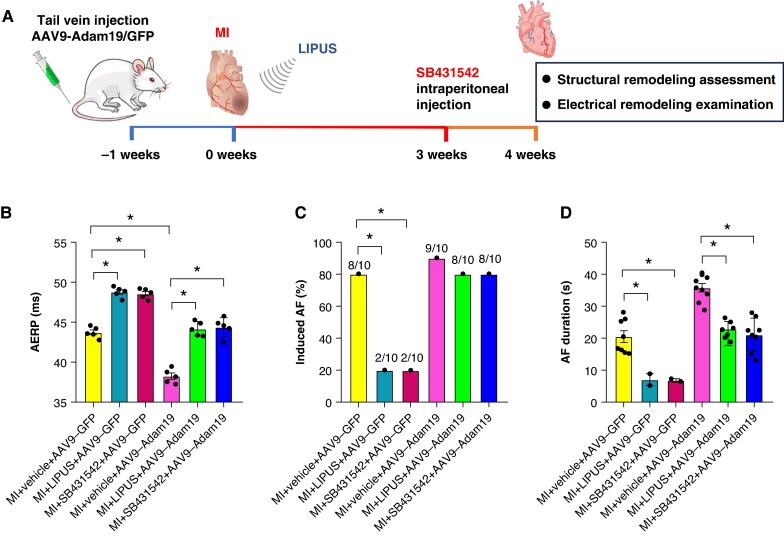
Activation of Adam19/TGF-β/Smad2/3 signalling counteracted the protective effect of LIPUS treatment on MI-induced atrial electrical remodelling. (*A*) Detailed experimental design flow chart. (*B*) Quantification of AERP (*n* = 5 per group). (*C*) Ratio of burst-induced AF and (*D*) duration of AF (*n* = 10 per group). *P < 0.05. Statistics: AF incidence by two-tailed Fisher’s exact test; AERP and AF duration by one-way ANOVA with Tukey’s multiple-comparisons test. Adam19, a disintegrin and metalloproteinase 19; AERP, atrial effective refractory period; AF, atrial fibrillation; LIPUS, low-intensity pulsed ultrasound; MI, myocardial infarction; TGF-β, transforming growth factor-β.

To further clarify whether the Adam19/TGF-β/Smad2/3 pathway involved in MI-induced AF. We also transfected the atrium with AAV9-Adam19 to overexpress Adam19. Additionally, SB431542, a special inhibitor of TGF-β, was administered in rats by intraperitoneal injection. And the detailed experimental design flow chart is shown in *Figure 10A*. Adam19 overexpression significantly reduced AERP in the MI + AAV9-Adam19 group compared to the MI + AAV9-GFP group, SB431542 treatment significantly prolonged AERP in the MI + AAV9-Adam19 group compared to the MI vehicle + Adam19 group, counteracted the worsening effect of AAV9-Adam19 (*Figure 10B*). Meanwhile, Adam19 overexpression has an increased trend of AF inducibility ratio compared to the MI group and offset the AF inhibition effect of LIPUS treatment, and SB431542 treatment significantly reduced AF duration compared to the MI + vehicle + AAV9-Adam19 group, counteracted the worsening effect of AAV9-Adam19 (*Figure 10C* and *D*).

### Activation of Adam19/TGF-β/Smad2/3 signalling abrogated the protective effect of low-intensity pulsed ultrasound treatment on myocardial infarction- induced atrial structural remodelling

**Figure 11:**
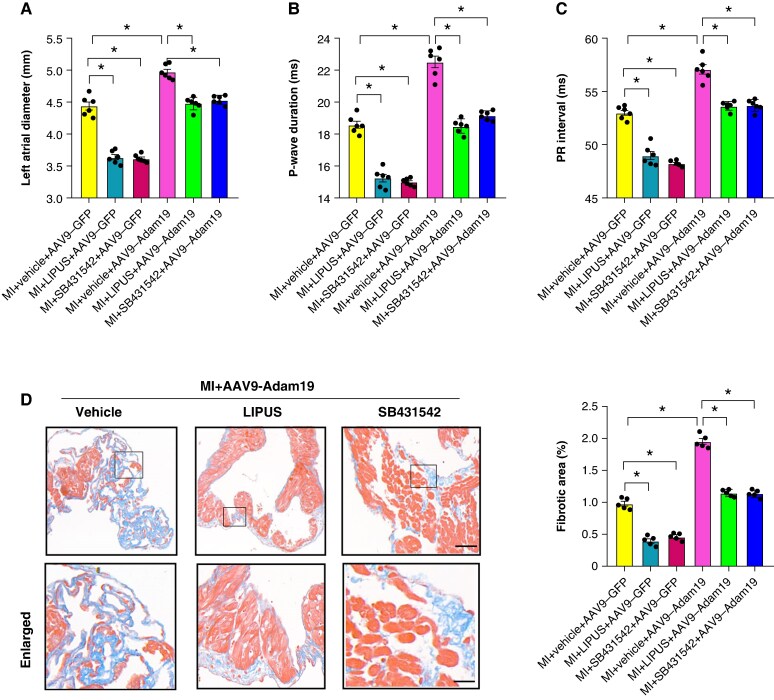
Activation of Adam19/TGF-β/Smad2/3 signalling abrogated the protective effect of LIPUS treatment on MI-induced atrial structural remodelling. (*A*) Quantification of the left atrial diameter (*n* = 6 per group). (*B*) Quantification of *P* wave duration and (*C*) duration of PR interval. (*n* = 6 per group). (*D*) Representative images of Masson-stained heart sections and quantification of percentage of the fibrotic area (*n* = 5 per group). *P < 0.05. Statistics: one-way ANOVA with Tukey’s multiple-comparisons test. Adam19, a disintegrin and metalloproteinase 19; LIPUS, low-intensity pulsed ultrasound; MI, myocardial infarction; TGF-β, transforming growth factor-β.

We also checked the effect of activation of Adam19/TGF-β/Smad2/3 signalling on MI-induced atrial structural remodelling. Adam19 overexpression significantly deteriorated MI-induced atrial enlargement, reflected by increased left atrial diameter, *P* wave duration, and PR interval, and Adam19 overexpression abrogated the reversal effect of LIPUS treatment or SB431542 treatment on MI-induced atrial enlargement (*Figure 11A–C*). Moreover, Adam19 overexpression significantly exacerbated MI-induced atrial fibrosis and abolished the antifibrotic effect of LIPUS treatment or SB431542 treatment (*Figure 11D*). Notably, overexpression of Adam19 did not abolish the left ventricle (LV) functional benefit of LIPUS (see Supplementary material online, *Figure S10*), and overexpression of Adam19 did not abolish the LV functional benefit of LIPUS on ventricular hypertrophy, fibrosis, cardiac function, and ventricular arrhythmia (VA) inducibility (see Supplementary material online, *Figure S10A*–*F*). Collectively, these data indicate a potential important role of Adam19/TGF-β/Smad2/3 signalling cascades in the antiarrhythmic effect of LIPUS.

## Discussion

This work is the first to show that LIPUS can curb post-MI AF. In healthy rats, 4 weeks of LIPUS left atrial structure, function, and electrophysiology unchanged, suggesting a favourable preliminary safety profile under the conditions tested. In MI rats, LIPUS attenuated electrical and structural remodelling and markedly reduced AF inducibility. RNA sequencing pointed to extracellular matrix pathways and revealed the potential effect of Adam19/TGF-β/Smad2/3 cascade in response to LIPUS therapy. Activation of Adam19/TGF-β/Smad2/3 worsened fibrosis and abolished LIPUS’s protection. The same anti-fibrotic, antiarrhythmic effects were reproduced in an independent ACh/CaCl₂ AF model. Collectively, these findings position LIPUS as a potentially safe, non-invasive strategy to attenuate AF after MI.

Structural remodelling is the basis for the occurrence of AF following post-MI.^13,14^ Atrial fibrosis and inflammation are acknowledged as critical contributors to the pathophysiology of AF. Enhanced levels of atrial fibrosis and inflammation are associated with slowed conduction, which creates a substrate for electrical reentry, ultimately leading to AF. LA fibrosis, characterized by abnormal proliferation of atrial fibroblasts and excessive extracellular matrix deposition, is the hallmark of structural remodelling of AF substrates.^15^ Sacubitril/valsartan treatment significantly decreased left atrium (LA) fibrosis and susceptibility to AF.^16^ Exploring new therapeutic tools and approaches to inhibit atrial fibrosis and atrial structural remodelling could inhibit AF post-MI. Accumulating evidence supported that LIPUS attenuates ventricular remodelling by stimulating angiogenesis and curbing hypertrophy, fibrosis, and inflammation,^7–9^ but its atrial effects have been unknown. We now provide the first evidence that LIPUS protects the atrium: in a post-MI model, it markedly attenuated atrial extracellular matrix (ECM) accumulation, mirroring its antifibrotic action in the ventricle. In addition, atrial inflammation also played an important role in AF post-MI. Pro-inflammatory cytokines such as IL-1β and IL-6 surge after MI, drive atrial inflammation, and further promote fibrosis through myofibroblast recruitment.^17–19^ Prior work shows that LIPUS blunts ventricular inflammation and remodelling.^20,21^ Consistent with previous research, our data show that LIPUS treatment significantly reduced atrial inflammation in the post-MI setting. These findings suggest that LIPUS may exert both anti-fibrotic and anti-inflammatory effects in the atria following MI.

In addition to structural remodelling, electrical remodelling also plays a pivotal role in the pathogenesis of AF. The reduction in AERP is known to create a substrate for conduction abnormalities and reentrant pathways, ultimately leading to AF. Our previous study indicated that shortened AERP facilitated AF induced by metabolic syndrome.^22^ This study found reduced AERP in the MI group, similar to previous reports.^23^ However, inconsistent with Liu *et al.*^17^ who reported that prolonged atrial ERP was found in the MI group, our findings found shortened AERP in the MI group. This difference may be due to the AERP that was measured in the early phase post-MI, as reported by Liu *et al.*,^17^ and the AERP was recorded at the chronic phase in our study. The impact of LIPUS on atrial electrical remodelling and AF susceptibility has only recently been investigated. Our findings indicate that LIPUS treatment can attenuate atrial electrical remodelling and reduce AF susceptibility by increasing the AERP. Furthermore, prolonged *P* wave and PR interval durations, which are associated with atrial enlargement and slowed atrial conduction, provide a conducive substrate for AF reentry.^22^ Low-intensity pulsed ultrasound could decrease *P* wave and PR interval durations while simultaneously increasing Cx43 expression and conduction velocity in the atrium. Electrical and structural remodelling significantly impedes impulse transmission and contributes to aberrant electrical signal propagation. Furthermore, a reduction in electrical connectivity, as evidenced by the loss of Cx43, the primary atrial gap junction protein, leads to desynchronized cardiac activation and contraction.^24^ Consequently, LIPUS could alleviate MI-induced atrial electrical remodelling through decreasing *P* wave and PR duration, prolonging AERP, and increasing Cx43 expression in the atrium. Consistent with previous reports,^17,25,26^ we observed a marked post-MI PR-interval prolongation. In our rat model, this delay likely reflects the effects of atrial ventricular (AV)-nodal ischaemia/fibrosis—mechanisms mitigated in contemporary human MI by right-coronary dominance and prompt reperfusion therapy. The absolute magnitude of PR change in rodents should thus be interpreted cautiously and not directly extrapolated to clinical practice.

RNA sequencing revealed Adam19 as the most prominently regulated gene after LIPUS in MI rat atria: it rose markedly post-MI but fell towards baseline with LIPUS, a pattern confirmed by western blotting. Adam19 contains several complex domains, including a pro-domain, metalloproteinase domain, disintegrin domain, cysteine-rich domain, epidermal growth factor-like domain, transmembrane domain, and cytoplasmic tail domain.^27^ It is broadly expressed (heart, lung, bone, brain, spleen, liver, skeletal muscle, kidney, testes) and is integral to embryo implantation, cardiovascular morphogenesis, and neurogenesis^28^ Loss-of-function studies show that Adam19-deficient mice develop ventricular septal defects and immature valves with poor remodelling of cardiac jelly,^12^  ^,29^ while ventricular pacing and dilatation progressively up-regulate Adam19.^30^ Its atrial role, however, was unknown. Here, AAV9-mediated Adam19 overexpression in the atrium aggravated fibrosis and heightened AF susceptibility, completely negating LIPUS protection. These findings indicate that LIPUS mitigates MI-induced atrial remodelling and AF primarily by down-regulating Adam19.

Growing evidence implicates Adam19 in fibrosis through the TGF-β/Smad2/3 axis. In idiopathic pulmonary fibrosis, TGF-β1 markedly elevates Adam19 mRNA, whereas silencing Adam19 curtails collagen deposition and lung scarring.^31^ A parallel mechanism operates in the kidney: TGF-β up-regulates Adam19, an effect attenuated by the Smad2/3 inhibitor SB525334, and high Adam19 expression accompanies profibrotic, pro-inflammatory renal decline.^32,33^ Moreover, Gao *et al.*^34^ revealed that Adam19 was closely related to the TGF-β1 pathway and cardiac fibrosis. The TGF-β/Smad2/3 signalling pathway regulates atrial fibrosis and AF.^35,36^ Consistent with this paradigm, we found that LIPUS lowers Adam19 and suppresses Smad2/3 activation, while AAV9-mediated Adam19 overexpression reactivates the pathway and abolishes LIPUS protection. Our observations align with Pan *et al.*^37^ who showed LIPUS-modulated extracellular matrix dynamics via TGF-β/Smad signalling in chondrocytes and together point to an Adam19/TGF-β/Smad2/3 interaction underlying LIPUS-mediated resistance to MI-induced AF. Prior studies report that LIPUS modulates multiple pathways—including increased M2 macrophage polarization,^38^ caveolin-1,^9^ β1-integrins,^9^ endothelial nitric oxide synthase (eNOS)/nitric oxide synthase (NOS),^39^ and mitogen-activated protein kinase (MAPK) signaling^40^—that could account for or contribute to observed effects. By contrast, in our study, left atrial RNA-seq (MI vs. MI + LIPUS) showed significant enrichment of extracellular matrix/collagen-assembly pathways (*Figure 7C*), concordant with attenuated fibrosis and consistent with the Adam19/TGF-β/Smad2/3 axis, suggesting that LIPUS primarily promotes atrial reverse remodelling via the ECM/TGF-β pathway. Caveolin-1/β1-integrin, eNOS, MAPK, and immune-cell polarization pathways were not significantly enriched at this time point, implying upstream positioning or temporal offset. Given atrial anatomical and biomechanical features, the phenotype appears predominantly antifibrotic. While our data support Adam19-driven augmentation of TGF-β/Smad2/3 signalling in post-MI atria, reports from other tissues indicate that TGF-β can transcriptionally induce ADAM19, consistent with a positive feed-forward loop.^32,41^ Accordingly, TGF-β blockade would be predicted to attenuate Adam19 induction/activation and thereby interrupt the loop; in our study, pathway-level inhibition (SB431542) normalized p-Smad2/3 and downstream ECM targets after Adam19 overexpression, but we did not quantify effects on Adam19 protein/activity, which remains to be resolved experimentally. Future studies will incorporate time-resolved and causal perturbations to define atrial–ventricular pathway specificity.

Atrial cardiomyopathy is a syndrome causing atrial structural, contractile, and electrophysiological abnormalities; it provides the substrate for AF and is further accelerated by AF. Goette *et al.*^42^ propose a three-stage classification integrating biomarkers, imaging, and electrophysiology; Remme *et al.*^43^ emphasize mechanism-guided precision therapy. We show that LIPUS down-regulates Adam19 and suppresses the TGF-β/Smad2/3 axis, exerting anti-inflammatory and anti-fibrotic effects, improving AERP, and reducing AF susceptibility. We recommend stage-stratified enrolment and composite reverse-remodelling endpoints—late gadolinium enhancement cardiovascular magnetic resonance (LGE-CMR) fibrosis burden, left atrial strain, electromechanical indices, and inflammation/fibrosis biomarkers.

Because LIPUS is a novel intervention, we first verified safety in healthy myocardium: hearts exposed at the same intensity showed no detectable structural, functional, or electrophysiological changes. To test efficacy beyond MI, we used an ACh/CaCl₂–induced AF model and found that LIPUS markedly attenuated atrial structural/electrical remodelling and reduced AF susceptibility. In the MI model, LIPUS improved LV systolic function and lowered ventricular arrhythmia susceptibility without significantly altering LV fibrosis at endpoint, suggesting that part of the atrial benefit could be secondary to improved ventricular performance. Collectively, these data support a context-dependent, multi-node framework with predominantly atrial-directed effects: in post-MI atria, an Adam19–TGF-β/Smad2/3 node plausibly mediates anti-remodelling benefits, whereas in ACh/CaCl₂ the benefit may reflect mitigation of I_K, ACh_-associated AERP shortening and improved Ca²⁺ handling. Low-intensity pulsed ultrasound pathways described in ventricular injury (anti-inflammatory, anti-oxidative, mitochondrial) may also indirectly stabilize atrial electrophysiology. Nonetheless, chamber-unique mechanisms and ventricular contributions cannot be excluded (no ventricular RNA-seq; limited ACh/CaCl₂ atrial molecular profiling). Prospective studies incorporating cholinergic and Ca²⁺ panels with autonomic readouts, followed by large-animal and early-phase clinical testing, are required to establish cross-model mechanistic robustness and translational relevance.

## Limitations

Several limitations warrant consideration. First, our mechanistic inference is MI-centric: although LIPUS likewise reduced AF and atrial remodelling in the ACh/CaCl₂ model, we did not perform atrial molecular validation in that model (Adam19, p-Smad2/3, and selected cholinergic/Ca²⁺-handling markers), which remains a priority for future work. Second, Adam19 causality *in vivo* remains unproven. In this cycle, we used cardiomyocyte-targeted AAV9 overexpression and pathway pharmacology (SB431542; BB-94 *in vitro*); a cell-specific loss of function was infeasible because atrial-selective, time-controlled rat tools were unavailable, global/cardiomyocyte-wide suppression could confound atrial readouts, available ADAM inhibitors lack Adam19 specificity, and a *de novo* AAV/CRISPRi programme would exceed our timeline and approvals. Accordingly, we interpret the data as consistent with modulation of an Adam19–TGF-β/Smad2/3 cascade and will pursue inducible and atrial- or cardiomyocyte-restricted Adam19 LOF and pathway-ordering studies. Third, while safety was assessed in healthy rats at structural, functional, and electrophysiological levels, we did not pre-specify a molecular neutrality panel in sham atria (Adam19, collagen-I/collagen-III/TGF-β, IL-1β/IL-6/TNF-α, Cx43), so transcript/protein neutrality remains to be documented before application to humans. Fourth, the Adam19/TGF-β/Smad2/3 axis likely represents one node within a broader remodelling network, and parallel pathways may also drive post-MI fibrosis and electrophysiological change possibly by feeding into TGF-β. Fifth, some atrial benefit may be secondary to improved LV systolic function with LIPUS; despite minimal endpoint changes in LV fibrosis or inflammation, a ventricular contribution cannot be excluded. Sixth, the electrophysiological assessment of this study was limited by the incomplete AERP measurement (about 50% of the hearts in each group did not meet the inclusion criteria, resulting in a smaller sample size than the AF induction test and reducing the estimation accuracy). Therefore, the relevant results were only exploratory, with insufficient statistical grasp (mostly nominally significant), and the extrapolation was limited. Finally, the MI-induced AF model has inherent limitations, and inter-species differences and tightly controlled experimental conditions constrain immediate translation, particularly to non-ischaemic AF, underscoring the need for validation in large-animal studies and phased human trials.

## Conclusions

In preclinical post-MI models, LIPUS attenuated atrial structural and electrical remodelling and lowered AF susceptibility, plausibly via modulation of an Adam19/TGF-β/Smad2/3 signalling cascade. These findings are promising but preliminary; priorities include determining whether the atrial effects are direct or secondary to ventricular remodelling and altered haemodynamics, confirming mechanisms across models, defining dose–response and safety, and validating efficacy and translational relevance in large-animal studies and early-phase trials before any clinical application.

## Supplementary Material

euaf258_Supplementary_Data

## Data Availability

The data underlying this article will be shared on reasonable request to the corresponding author.

## References

[1] Schmitt J, Duray G, Gersh BJ, Hohnloser SH. Atrial fibrillation in acute myocardial infarction: a systematic review of the incidence, clinical features and prognostic implications. Eur Heart J 2009;30:1038–45. 19109347 10.1093/eurheartj/ehn579

[2] Frederiksen TC, Dahm CC, Preis SR, Lin H, Trinquart L, Benjamin EJ et al The bidirectional association between atrial fibrillation and myocardial infarction. Nat Rev Cardiol 2023;20:631–44. 37069297 10.1038/s41569-023-00857-3 PMC11380523

[3] Garg L, Agrawal S, Agarwal M, Shah M, Garg A, Patel B et al Influence of atrial fibrillation on outcomes in patients who underwent primary percutaneous coronary intervention for ST-segment elevation myocardial infarction. Am J Cardiol 2018;121:684–9. 29394997 10.1016/j.amjcard.2017.12.003

[4] Jabre P, Roger VL, Murad MH, Chamberlain AM, Prokop L, Adnet F et al Mortality associated with atrial fibrillation in patients with myocardial infarction: a systematic review and meta-analysis. Circulation 2011;123:1587–93. 21464054 10.1161/CIRCULATIONAHA.110.986661 PMC3082773

[5] Wang J, Yang YM, Zhu J. Mechanisms of new-onset atrial fibrillation complicating acute coronary syndrome. Herz 2015;40:18–26. 25352243 10.1007/s00059-014-4149-3

[6] El-Shetry M, Mahfouz R, Frere AF, Abdeldayem M. The interplay between atrial fibrillation and acute myocardial infarction. Br J Hosp Med (Lond) 2021;82:1–9. 10.12968/hmed.2020.0584 33646024

[7] Zhao K, Zhang J, Xu T, Yang C, Weng L, Wu T et al Low-intensity pulsed ultrasound ameliorates angiotensin II-induced cardiac fibrosis by alleviating inflammation via a caveolin-1-dependent pathway. J Zhejiang Univ Sci B 2021;22:818–38. 34636186 10.1631/jzus.B2100130 PMC8505463

[8] Monma Y, Shindo T, Eguchi K, Kurosawa R, Kagaya Y, Ikumi Y et al Low-intensity pulsed ultrasound ameliorates cardiac diastolic dysfunction in mice: a possible novel therapy for heart failure with preserved left ventricular ejection fraction. Cardiovasc Res 2021;117:1325–38. 32683442 10.1093/cvr/cvaa221

[9] Shindo T, Ito K, Ogata T, Hatanaka K, Kurosawa R, Eguchi K et al Low-intensity pulsed ultrasound enhances angiogenesis and ameliorates left ventricular dysfunction in a mouse model of acute myocardial infarction. Arterioscler Thromb Vasc Biol 2016;36:1220–9. 27079882 10.1161/ATVBAHA.115.306477

[10] Meng Q, Bao D, Liu S, Huang J, Guo M, Dai B et al ADAM metallopeptidase domain 19 promotes skin fibrosis in systemic sclerosis via neuregulin-1. Mol Med 2024;30:269. 39716051 10.1186/s10020-024-01047-8 PMC11665244

[11] Manso AM, Elsherif L, Kang SM, Ross RS. Integrins, membrane-type matrix metalloproteinases and ADAMs: potential implications for cardiac remodeling. Cardiovasc Res 2006;69:574–84. 16253214 10.1016/j.cardiores.2005.09.004

[12] Kurohara K, Komatsu K, Kurisaki T, Masuda A, Irie N, Asano M et al Essential roles of Meltrin beta (ADAM19) in heart development. Dev Biol 2004;267:14–28. 14975714 10.1016/j.ydbio.2003.10.021

[13] Hwang HJ, Ha JW, Joung B, Choi EH, Kim J, Ahn MS et al Relation of inflammation and left atrial remodeling in atrial fibrillation occurring in early phase of acute myocardial infarction. Int J Cardiol 2011;146:28–31. 19570585 10.1016/j.ijcard.2009.05.065

[14] Wang Q, Yu Y, Zhang P, Chen Y, Li C, Chen J et al The crucial role of activin A/ALK4 pathway in the pathogenesis of Ang-II-induced atrial fibrosis and vulnerability to atrial fibrillation. Basic Res Cardiol 2017;112:47. 28639003 10.1007/s00395-017-0634-1

[15] Surinkaew S, Aflaki M, Takawale A, Chen Y, Qi XY, Gillis MA et al Exchange protein activated by cyclic-adenosine monophosphate (Epac) regulates atrial fibroblast function and controls cardiac remodelling. Cardiovasc Res 2019;115:94–106. 30016400 10.1093/cvr/cvy173 PMC6302269

[16] Suo Y, Yuan M, Li H, Zhang Y, Li Y, Fu H et al Sacubitril/valsartan improves left atrial and left atrial appendage function in patients with atrial fibrillation and in pressure overload-induced mice. Front Pharmacol 2019;10:1285. 31736759 10.3389/fphar.2019.01285 PMC6830387

[17] Liu M, Li W, Wang H, Yin L, Ye B, Tang Y et al CTRP9 ameliorates atrial inflammation, fibrosis, and vulnerability to atrial fibrillation in post-myocardial infarction rats. J Am Heart Assoc 2019;8:e013133. 31623508 10.1161/JAHA.119.013133 PMC6898814

[18] Harada M, Van Wagoner DR, Nattel S. Role of inflammation in atrial fibrillation pathophysiology and management. Circ J 2015;79:495–502. 25746525 10.1253/circj.CJ-15-0138 PMC4457364

[19] Fukui A, Takahashi N, Nakada C, Masaki T, Kume O, Shinohara T et al Role of leptin signaling in the pathogenesis of angiotensin II-mediated atrial fibrosis and fibrillation. Circ Arrhythm Electrophysiol 2013;6:402–9. 23406575 10.1161/CIRCEP.111.000104

[20] Cao Q, Liu L, Hu Y, Cao S, Tan T, Huang X et al Low-intensity pulsed ultrasound of different intensities differently affects myocardial ischemia/reperfusion injury by modulating cardiac oxidative stress and inflammatory reaction. Front Immunol 2023;14:1248056. 37744362 10.3389/fimmu.2023.1248056 PMC10513435

[21] Yang H, Hu Y, Kong B, Zhou Y, Shuai W. Low-intensity pulsed ultrasound treatment mitigates ventricular arrhythmias via inhibiting microglia-mediated neuroinflammation in heart failure rat model. Int Immunopharmacol 2024;126:111317. 38048669 10.1016/j.intimp.2023.111317

[22] Yang HJ, Kong B, Shuai W, Zhang JJ, Huang H. Shensong Yangxin attenuates metabolic syndrome-induced atrial fibrillation via inhibition of ferroportin-mediated intracellular iron overload. Phytomedicine 2022;101:154086. 35421806 10.1016/j.phymed.2022.154086

[23] Ye T, Liu X, Qu C, Zhang C, Fo Y, Guo Y et al Chronic inhibition of the sigma-1 receptor exacerbates atrial fibrillation susceptibility in rats by promoting atrial remodeling. Life Sci 2019;235:116837. 31493481 10.1016/j.lfs.2019.116837

[24] Guo YH, Yang YQ. Atrial fibrillation: focus on myocardial connexins and gap junctions. Biology (Basel) 2022;11:489. 35453689 10.3390/biology11040489 PMC9029470

[25] Qiu H, Liu W, Lan T, Pan W, Chen X, Wu H et al Salvianolate reduces atrial fibrillation through suppressing atrial interstitial fibrosis by inhibiting TGF-beta1/Smad2/3 and TXNIP/NLRP3 inflammasome signaling pathways in post-MI rats. Phytomedicine 2018;51:255–65. 30466624 10.1016/j.phymed.2018.09.238

[26] Hiram R, Xiong F, Naud P, Xiao J, Sosnowski DK, Le Quilliec E et al An inflammation resolution-promoting intervention prevents atrial fibrillation caused by left ventricular dysfunction. Cardiovasc Res 2024;120:345–59. 38091977 10.1093/cvr/cvad175 PMC10981525

[27] Qi B, Newcomer RG, Sang QX. ADAM19/adamalysin 19 structure, function, and role as a putative target in tumors and inflammatory diseases. Curr Pharm Des 2009;15:2336–48. 19601835 10.2174/138161209788682352

[28] Li J, Perfetto M, Neuner R, Bahudhanapati H, Christian L, Mathavan K et al Xenopus ADAM19 regulates Wnt signaling and neural crest specification by stabilizing ADAM13. Development 2018;145:dev158154. 29540504 10.1242/dev.158154 PMC5963864

[29] Zhou HM, Weskamp G, Chesneau V, Sahin U, Vortkamp A, Horiuchi K et al Essential role for ADAM19 in cardiovascular morphogenesis. Mol Cell Biol 2004;24:96–104. 14673146 10.1128/MCB.24.1.96-104.2004 PMC303363

[30] Doggen K, Ray L, Mathieu M, Mc Entee K, Lemmens K, De Keulenaer GW. Ventricular ErbB2/ErbB4 activation and downstream signaling in pacing-induced heart failure. J Mol Cell Cardiol 2009;46:33–8. 19010331 10.1016/j.yjmcc.2008.10.010

[31] Keating DT, Sadlier DM, Patricelli A, Smith SM, Walls D, Egan JJ et al Microarray identifies ADAM family members as key responders to TGF-beta1 in alveolar epithelial cells. Respir Res 2006;7:114. 16948840 10.1186/1465-9921-7-114 PMC1569837

[32] Ramdas V, McBride M, Denby L, Baker AH. Canonical transforming growth factor-beta signaling regulates disintegrin metalloprotease expression in experimental renal fibrosis via miR-29. Am J Pathol 2013;183:1885–96. 24103556 10.1016/j.ajpath.2013.08.027 PMC4188136

[33] Melenhorst WB, van den Heuvel MC, Timmer A, Huitema S, Bulthuis M, Timens W et al ADAM19 expression in human nephrogenesis and renal disease: associations with clinical and structural deterioration. Kidney Int 2006;70:1269–78. 16900093 10.1038/sj.ki.5001753

[34] Gao QY, Zhang HF, Chen ZT, Li YW, Wang SH, Wen ZZ et al Construction and analysis of a ceRNA network in cardiac fibroblast during fibrosis based on in vivo and in vitro data. Front Genet 2020;11:503256. 33552116 10.3389/fgene.2020.503256 PMC7859616

[35] Li X, Zhu F, Meng W, Zhang F, Hong J, Zhang G et al CYP2J2/EET reduces vulnerability to atrial fibrillation in chronic pressure overload mice. J Cell Mol Med 2020;24:862–74. 31749335 10.1111/jcmm.14796 PMC6933320

[36] Wu Y, Luo J, Song X, Gu W, Wang S, Hao S et al Irisin attenuates angiotensin II-induced atrial fibrillation and atrial fibrosis via LOXL2 and TGFbeta1/Smad2/3 signaling pathways. Iran J Basic Med Sci 2023;26:717–24. 37275755 10.22038/IJBMS.2023.68639.14967 PMC10237168

[37] Pan YL, Ma Y, Guo Y, Tu J, Guo GP, Ma SM et al Effects of Clematis chinensis osbeck mediated by low-intensity pulsed ultrasound on transforming growth factor-beta/Smad signaling in rabbit articular chondrocytes. J Med Ultrason (2001) 2019;46:177–86. 30659392 10.1007/s10396-018-0920-z

[38] Xu Z, Li S, Wan L, Hu J, Lu H, Zhang T. Role of low-intensity pulsed ultrasound in regulating macrophage polarization to accelerate tendon-bone interface repair. J Orthop Res 2023;41:919–29. 36203341 10.1002/jor.25454

[39] Nakata T, Shindo T, Ito K, Eguchi K, Monma Y, Ichijo S et al Beneficial effects of low-intensity pulsed ultrasound therapy on right ventricular dysfunction in animal models. JACC Basic Transl Sci 2023;8:283–97. 37034290 10.1016/j.jacbts.2022.08.010 PMC10077125

[40] Ling L, Wei T, He L, Wang Y, Wang Y, Feng X et al Low-intensity pulsed ultrasound activates ERK1/2 and PI3K-Akt signalling pathways and promotes the proliferation of human amnion-derived mesenchymal stem cells. Cell Prolif 2017;50:e12383. 28940899 10.1111/cpr.12383 PMC6529069

[41] Chan MW, Huang YW, Hartman-Frey C, Kuo CT, Deatherage D, Qin H et al Aberrant transforming growth factor beta1 signaling and SMAD4 nuclear translocation confer epigenetic repression of ADAM19 in ovarian cancer. Neoplasia 2008;10:908–19. 18714391 10.1593/neo.08540 PMC2517635

[42] Goette A, Corradi D, Dobrev D, Aguinaga L, Cabrera JA, Chugh SS et al Atrial cardiomyopathy revisited-evolution of a concept: a clinical consensus statement of the European Heart Rhythm Association (EHRA) of the ESC, the Heart Rhythm Society (HRS), the Asian Pacific Heart Rhythm Society (APHRS), and the Latin American Heart Rhythm Society (LAHRS). Europace 2024;26:euae204. 39077825 10.1093/europace/euae204 PMC11431804

[43] Remme CA, Heijman J, Gomez AM, Zaza A, Odening KE. 25 years of basic and translational science in EP Europace: novel insights into arrhythmia mechanisms and therapeutic strategies. Europace 2023;25:euad210. 37622575 10.1093/europace/euad210 PMC10450791

