# Supplementary material for: Low-intensity pulsed ultrasound attenuates atrial remodelling and atrial fibrillation after myocardial infarction: an experimental pre-clinical study
Source: Europace. 2025 Oct 9;27(10):euaf258. doi: 10.1093/europace/euaf258 (PMC12559852; doi:10.1093/europace/euaf258)
Supplement: euaf258_Supplementary_Data [file euaf258_supplementary_data.docx]

**Supplementary Material**

**1 Supplementary Methods**

**1.1 Animals and treatment**

100-male Sprague-Dawley rats weighing 250-300g were provided by Renmin Hospital of Wuhan University’s animal experiment center. This project was approved by the Animal Care and Use Committee of Renmin Hospital of Wuhan University (WDRM#20190429). The animal experiments were carried out according to the Guide for the Care and Use of Laboratory Animals published by the U.S. National Institutes of Health (NIH Publication No. 85-23, revised 2011). Rats were housed in conditions with temperature of 22±2°C and a 12:12-h light/dark cycle, ad libitum food, and tap water. The rats were randomly assigned into five groups, each consisting of 20 animals: the control group underwent a sham operation (sham), the sham group received low-intensity pulsed ultrasound (LIPUS) treatment (sham+LIPUS), the healthy rats received LIPUS treatment (control+LIPUS) group, the MI group, and the MI group treated with LIPUS (MI+LIPUS). Prior to establishing the MI model, all rats underwent a week of acclimatization with adapted feeding.

To overexpress Adam19 in rats, the rats were injected with adeno-associated virus 9 (AAV9) vectors carrying the Adam19 transcription factor under a cTnT promoter AAV9 expressing Adam19 (AAV9-Adam19) or green fluorescent protein (AAV9-GFP) (Genechem Co., Ltd.; Shanghai, China) via tail vein with 5 × 10^10^ viral genome particles per rat. Rats received an injection of AAV9-GFP or AAV9-Adam19 four week before MI model. To further clarify the potential effect of TGF-β/Smad2/3 signaling on LIPUS treatment, SB431542 (5 mg/kg, S1067, Selleckchem), a special inhibitor of TGF-β was administered daily in rats by intraperitoneal injection for 1-week [1, 2]. All experimental procedures were according to the guidance of Guidelines for the Care and Use of Laboratory Animals published by the US National Institutes of Health and were approved by the Animal Care and Use Committee of Renmin Hospital of Wuhan University.

**1.2 MI rat model**

The rat model of MI was carried out in accordance with prior reports.^10^ Rats were anaesthetized using pentobarbital sodium (40 mg/kg), and then they were given room air ventilation using a respirator. A thoracotomy and occlusion of the left anterior descending (LAD) coronary artery were performed on the rats in the MI groups. Two-lead ECG monitoring of ST-segment elevation. The rats in the sham group were threaded but not ligated at the same time. Penicillin (200,000 IU) was administered intramuscularly to all rats twice daily for one week.

**1.3 Acetylcholine (ACh)/calcium chloride (CaCl_2_)-induced AF rat model**

To further clarify the potential effect of LIPUS on AF inducibility, another AF rat model was produced. The rat model of AF was induced by ACh (66 μg/mL)-CaCl_2_ (50 mg/mL) at a dose of 0.1 mL/100 g via tail vein injection for 4-weeks [3, 4]. Eighty Male Sprague-Dawley rats were randomly assigned to four groups, as follows: control group: received 1 ml/kg isotonic saline; AF group: received 1 ml/kg induction mixture of ACh/CaCl_2_; control+LIPUS group and AF+LIPUS group. Following the tail vein injection of ACh/CaCl_2_, the rats in the control+LIPUS and AF+LIPUS group received LIPUS treatment daily (20 minutes three times per day under inhalation anesthesia) for 4-weeks.

**1.4 LIPUS treatment**

LIPUS (Chongqing Haifu Medical Technology Co., China) is a specific type of ultrasound that delivers at a low intensity and outputs in the mode of pulsed waves, and LIPUS could be transmitted through a medium to cells and tissues as high-frequency acoustic waves. It is generally generated by a transducer converting electrical power to mechanical energy through the mechanism of piezoelectricity. The periodic mechanical sound waves of LIPUS can cause vibrations and collisions by transmission through the medium. Compared to the high-intensity ultrasound used for tissue heating, LIPUS primarily delivers non-thermal effects, including microbubbles and microjets induced by cavitation, acoustic streaming, and mechanical stimulation, and so on [5, 6]. Except for its biophysical effects, LIPUS has been shown to exhibit beneficial biological effects in a number of previous studies [7-9]. The schematic diagram of LIPUS for treatments as shown in the ***Supplementary Figure S11.*** The detailed procedure is as follows: under mild inhalational anesthesia with isoflurane (0.5–1.0%), low-intensity pulsed ultrasound treatment was delivered directly trans-thoracically to the heart. The following LIPUS parameters were chosen based on earlier research: 1.0 MHz ultrasonic frequency, 10 Hz pulse repeat frequency, 20% duty cycle, and 30 mW/cm^2^ output intensity. For four weeks following the conclusion of the LAD operation, the rats in the sham+LIPUS and MI+LIPUS groups received LIPUS treatment daily (20 minutes three times per day under inhalation anesthesia). A low‐frequency ultrasound probe was attached to the chest of the rat, and an ultrasound coupling agent was applied. The LIPUS therapy was administered to the MI group and sham group 24-hours after the LAD or sham operation, however with the same anesthesia and probe application without electrical stimulation.

**1.5 Cell culture and LIPUS treatment**

The mouse myocardial originated HL‐1 cells were purchased from the cell bank of Chinese Academy of Science. HL‐1 cells were subjected to oxygen‐glucose deprivation (OGD) to mimic myocardial ischaemia in vitro. Briefly, the full‐growth medium was changed to serum‐free and glucose‐free DMEM, and then, cells were moved to an incubator containing 95% N_2_ and 5% CO_2_ at 37℃ for 3 h or the indicated time‐points. Control cells were kept in normal medium under normal conditions. To further verify the potential effect of LIPUS on Adam19/TGF-β/Smad2/3 signaling cascades, we added a cell-autonomous HL-1 atrial myocyte OGD model with six groups: PBS, OGD, OGD+LIPUS, OGD+LIPUS+Ad-Adam19, OGD+LIPUS+Ad-Adam19+BB-94 (a specific inhibitor of matrix metalloproteinases), and OGD+LIPUS+Ad-Adam19+SB431542 (a specific inhibitor of TGF-β). Initially, the cells were synchronized for 24 hours, followed by transfected with either Ad-Adam19 (Genechem Co., Ltd.; Shanghai, China) or control Ad-EV (empty vector) for 24 hours according to the manufacturers' instructions. Following Ad-Adam19 transfection, the cells were exposed to either OGD or phosphate-buffered saline (PBS). Then, HL‐1 cells were treated with LIPUS for 24-hours. Subsequently, the HL‐1 cells were treated with BB-94, a specific inhibitor of matrix metalloproteinases (20 μM) for 48 hours [10] or SB431542, a specific inhibitor of TGF-β (5 μM) for 48 hours [11].

The LIPUS exposure device (Chongqing Haifu Medical Technology Co., Ltd., Chongqing, China) comprises an array of five transducers (34.8 mm for circular diameter), which is specifically drafted for a 6-well culture plate. A 6-well plate, which was sealed with parafilm (a thickness of 1.5 mm) was deposited on the transducers with a thin layer of ultrasound coupling agent (about 1.5 mm thick) between them. The parameters of the LIPUS device were intensity of 120mW/cm^2^(spatial-average temporal-average intensity), the duty cycle of 20%, and pulse repetition frequency of 1 kHz; four frequencies (0.5, 1.0, 1.5, and 2.0 MHz) were used and the peak pressure was about 0.174MPa [12].

**1.6 Echocardiography assessment**

Echocardiography was performed to evaluate cardiac function at the end of the 4-week LIPUS treatment. In brief, echocardiography was performed under continuous anesthesia with 1.5% to 2% isoflurane, using a Mylab30CV (ESAOTE) ultrasound system with a 15MHz probe. Cardiac measurements included examination of left atrial dimension (LAD), left ventricular ejection fraction (LVEF), and fractional shortening (LVFS).

**1.7 *In vivo* Electrophysiological Study**

In brief, rats were anaesthetised with pentobarbital sodium (40 mg/kg i.p.; Sigma), intubated, and mechanically ventilated with a volume-controlled rodent ventilator. Following a midline sternotomy, the heart was exposed, and a bipolar silver-wire electrode (1 mm tip spacing) was secured to the right atrial appendage. Based on earlier research, the atrial effective refractory period (AERP) was carried out [13, 14].

According to our prior research, AF inducibility was carried out [14]. Protocols for burst pacing were used to assess AF susceptibility. All rat groups were subjected to three seconds of burst RA pacing with twice the threshold voltage (cycle lengths of 50 ms, pulse durations of 10 ms). A rapid irregular atrial rhythm with irregular R-R intervals lasting at least one second was deemed to be AF. From the end of burst pacing until the first P wave discovered following the rapid irregular atrial beat, the duration of AF was calculated. After the *In vivo* electrophysiological study, rats were killed in an open-chest procedure to access the heart.

**1.8 Electrocardiogram (ECG) recording**

The rats underwent anesthesia with pentobarbital sodium (40 mg/kg, i.p., Sigma) following a 4-week LIPUS therapy. ECGs were regularly captured in accordance with our earlier research [14]. The length of the P-wave and the PR intervals were then measured and examined.

**1.9 Histological analysis**

Embedded heart tissues were cut into 5μm sections. According to the manufacturers' instructions, Masson trichrome staining is used to assess the atrial fibrosis and Wheat Germ Agglutinin (WGA, L4895, Sigma Aldrich, USA) to assess atrial hypertrophy, according to the manufacturers’ instructions. The extent of atrial fibrosis and hypertrophy were assessed using Image J software (NIH Image, Bethesda, MD, USA).

**1.10 Immunofluorescence staining**

For immunofluorescence, paraffin slices were also employed, and atrial slides were stained with connexin 43 (Cx43) and CD68-specific primary antibodies. 4'6-diamidino-2-phenylindole (Sigma-Aldrich) was used to stain the nuclei. The images were captured using a Tokyo, Japan-made OLYMPUS BX51 fluorescent microscope. Each atrium sample-stained sections were examined and captured on camera using a microscopy (200x magnification).

**1.11 RNA sequencing (RNA-seq)**

Total RNA was extracted from atrial tissue using TRIzol™ Reagent (Invitrogen) and subjected to the GenePharma (Shanghai, China) for RNA-seq. Differential expression genes (DEGs) were considered significantly different when the log2FoldChange ≥1 or ≤−1, and the p < 0.05. The selected DEGs were cluster analyzed and a heat map was drawn in MultiExperiment Viewer software. These data are publicly available (BioProject ID PRJNA1069379).

**1.12 RT-qPCR**

Total RNA was extracted from cardiac tissues or cells using Trizol reagent (Invitrogen). First-strand cDNA was synthesized from total RNA using Prime Script™ RT Master Mix (Takara, Tokyo, Japan). RT-qPCR was performed in a 25μL reaction on the CFX96 Real-Time PCR Detection System (Bio-Rad Laboratories), including 0.4μmol/L primers, 50 ng of cDNA, and 12.5μL TB Green Premix Ex Taq II (Takara). The expression levels of target genes were normalized to the expression levels of beta-actin, which was considered as an endogenous internal control. The primer sequences were as exhibited in ***Supplementary Table S1 and Supplementary Table S2***.

**1.13 Western blotting**

As previously described, western blotting was used to assess protein expression levels [12]. Adam19 (1:1000, #AP9815c, Abcepta), Cx43 (1:1000, #ab314908, Abcam), TGF-β (1:1000, #21898-1-AP, Proteintech Group), p-Smad2 (1:500, #18338, CST), T-Smad2 (1:2000, #5339, CST), p-Smad3 (1:500, #9520, CST) and T-Smad3 (1:2000, #9523, CST), GAPDH (1:10000, #ab181602, Abcam) are the primary antibodies used in this manuscript. The amounts of total protein were adjusted to GAPDH.

**1.14 Statistical analysis**

Statistical analyses were conducted using GraphPad Prism version 7.0 and SPSS version 23.0. Data are presented as mean ± standard error of the mean (SEM) or as percentages. Sample sizes are specified in the corresponding figure legends. For datasets exhibiting a Gaussian distribution, comparisons between two groups were performed using an unpaired Student’s t-test with the assumption of equal variances, or with Welch’s correction for unequal variances. For non-Gaussian datasets, the Wilcoxon (Mann-Whitney U) test was employed for non-parametric statistical comparisons. Categorical data were analyzed using Fisher’s exact test. A p-value of less than 0.05 was considered to indicate statistical significance.

**2 Supplementary figures**


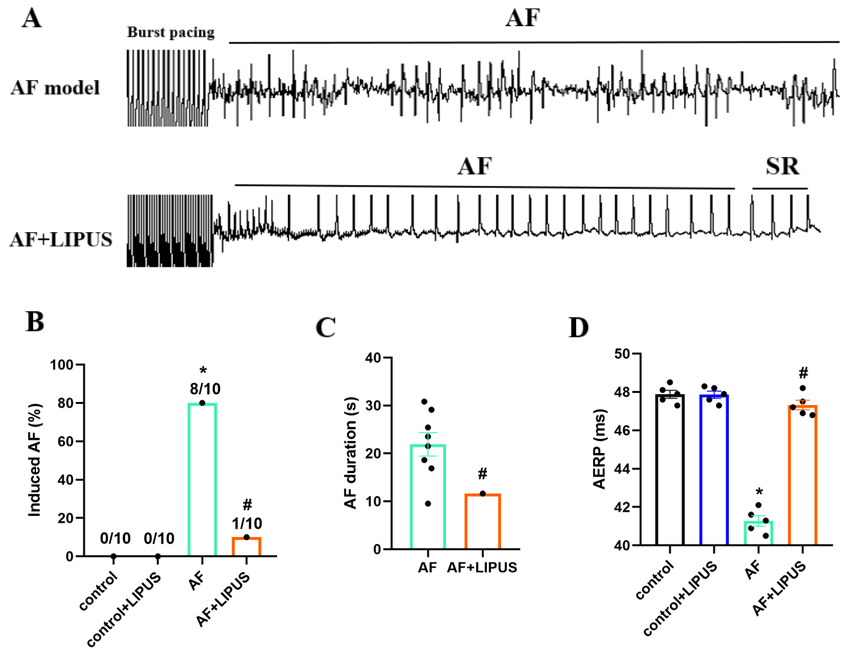


**Supplementary Figure S1. LIPUS treatment alleviated ACh/CaCl_2_-induced AF susceptibility. (A)** Representative examples of AF induced by burst-stimulating in AF group and AF+LIPUS group. **(B)** Ratio of burst-induced AF and **(C)** duration of AF (n = 10 per group). **(D)** Quantification of AERP. **P* < 0.05 vs. control group. #*P* < 0.05 vs. AF group. LIPUS = low-intensity pulsed ultrasound; AF = atrial fibrillation; AERP = atrial effective refractory period.


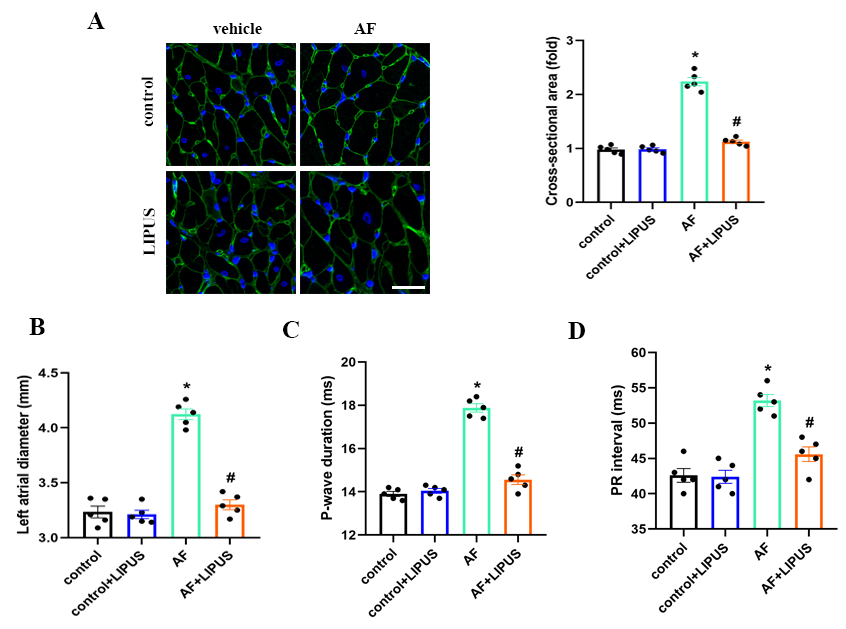
**Supplementary Figure S2. LIPUS therapy attenuated ACh/CaCl_2_-induced atrial enlargement. (A)** Representative WGA staining in atrial and quantitative results cross-sectional area (n = 5). **(B)** Quantification of left atrial diameter (n = 5). **(C)** Quantification of P-wave duration and **(D)** duration of PR interval. (n = 5 per group). **P* < 0.05 vs. control group. #*P* < 0.05 vs. AF group. WGA = wheat germ agglutinin.


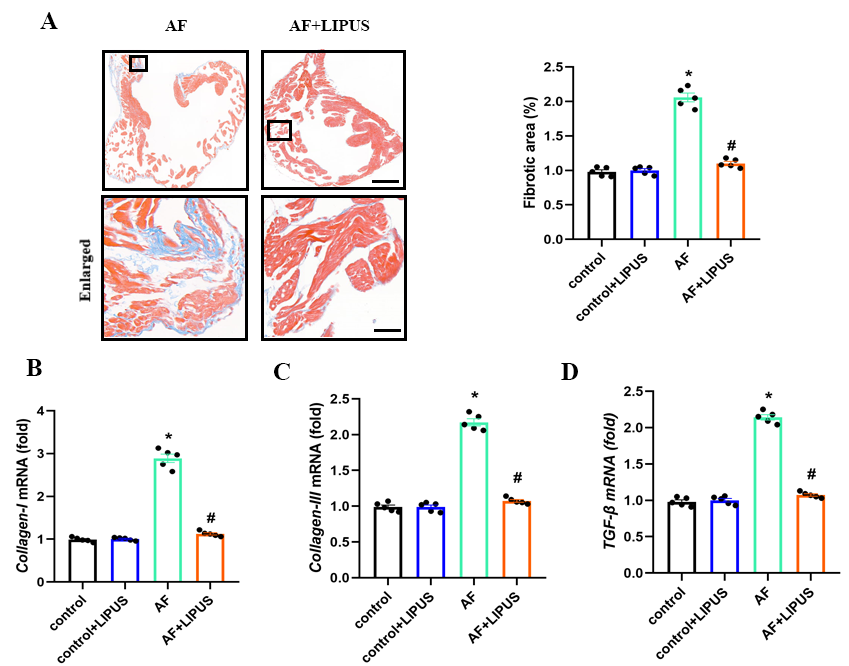
**Supplementary Figure S3. LIPUS therapy ameliorates atrial fibrosis induced by ACh/CaCl_2_. (A)** Representative images of Masson-stained heart sections and quantification of percentage of the fibrotic area (n = 5 per group). **(B-D)** Quantification of mRNA levels of *collagen-I, collagen-III* and *TGF-β* (n = 5 per group). **P* < 0.05 vs. control group. #*P* < 0.05 vs. AF group. TGF-β = transforming growth factor-β.


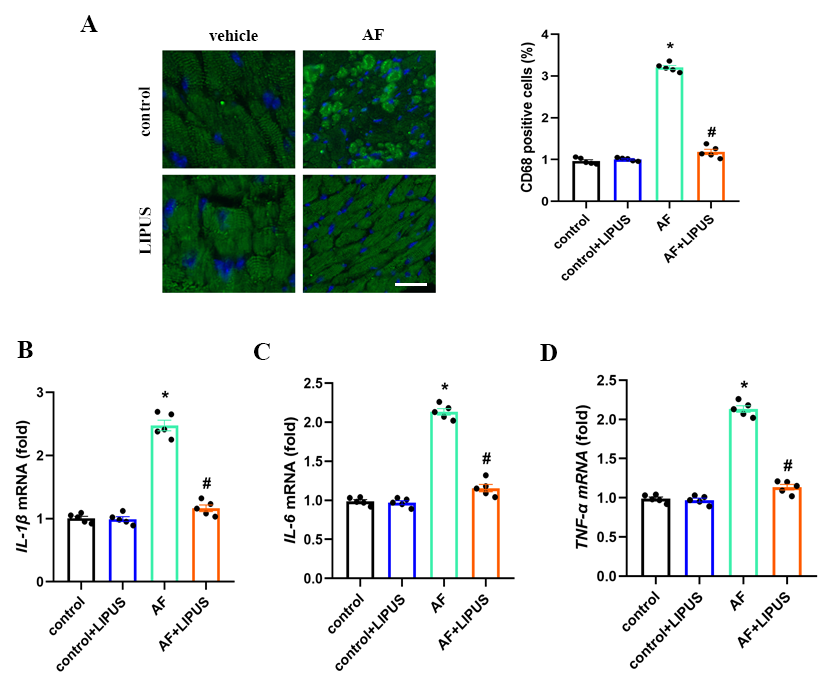


**Supplementary Figure S4. LIPUS therapy ameliorates atrial inflammation induced by ACh/CaCl_2_. (A)** Representative CD68 immunofluorescence and quantitative results of CD68-positive cells (n = 5 per group). **(B-D)** Quantification of mRNA levels of *IL-1β, IL-6* and *TNF-α* (n = 5 per group). **P* < 0.05 vs. control group. #*P* < 0.05 vs. AF group. IL-1β = Interleukin 1β; IL-6 = Interleukin 6; TNF-α = tumor necrosis factor α.


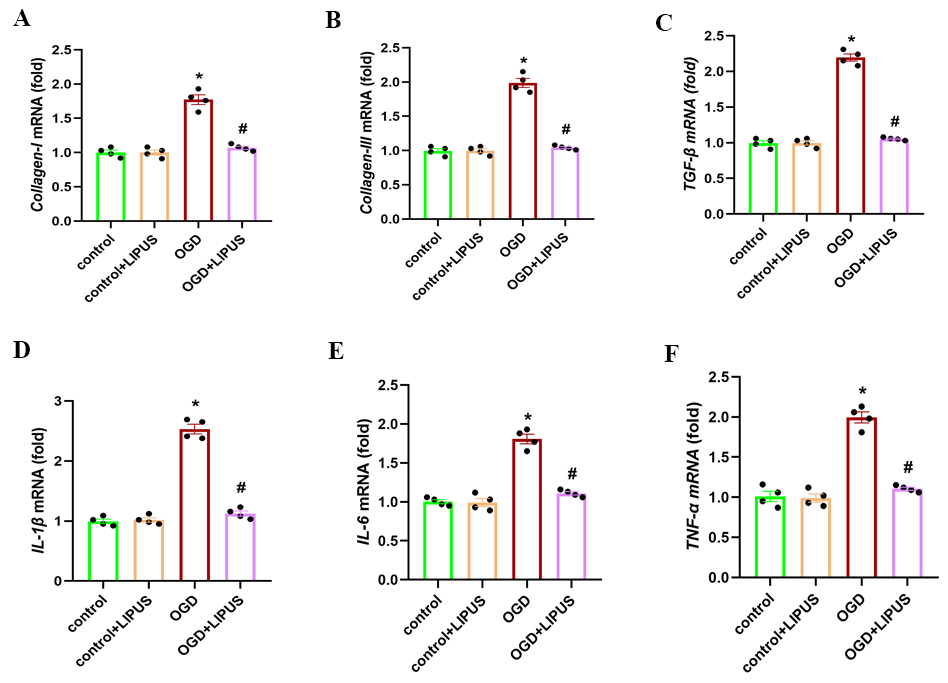


**Supplementary Figure S5. LIPUS therapy ameliorates cardiomyocyte fibrosis and inflammation induced by OGD in HL-1 cells. (A-C)** Quantification of mRNA levels of *collagen-I, collagen-III* and *TGF-β* (n = 4 per group). **(D-F)** Quantification of mRNA levels of *IL-1β, IL-6* and *TNF-α* (n = 4 per group). **P* < 0.05 vs. control group. #*P* < 0.05 vs. OGD group. OGD = oxygen‐glucose deprivation.


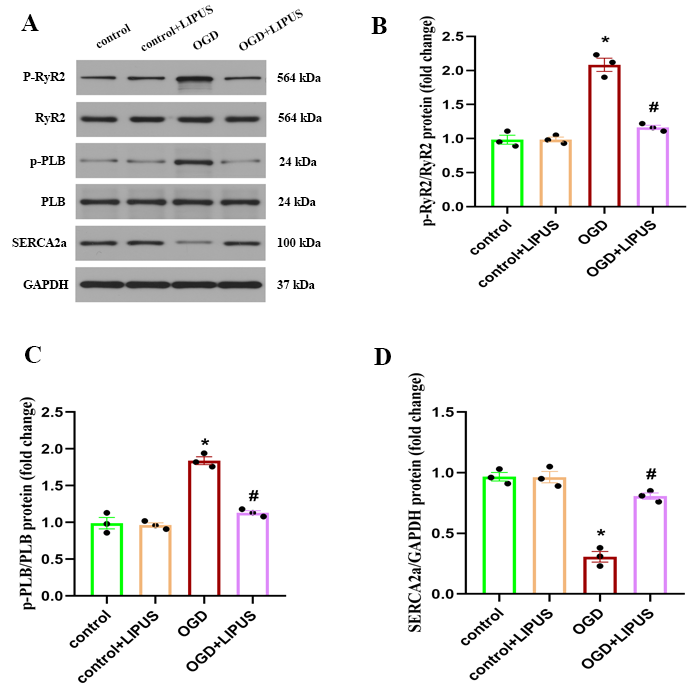


**Supplementary Figure S6. LIPUS therapy attenuated ACh/CaCl_2_-induced atrial electrical remodeling. (A)** Representative western blot and statistical results of p-RyR2/RyR2, p-PLB/PLB and SERCA2a protein expression (n = 3). **P* < 0.05 vs. control group. #*P* < 0.05 vs. AF group. SERCA2a = Ca^2+^-ATPase 2a; PLB = phospholamban; RyR2 = ryanodine receptor 2.


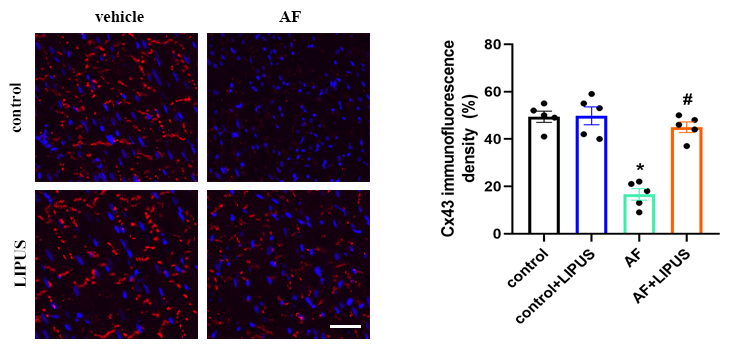


**Supplementary Figure S7. LIPUS treatment regulated ACh/CaCl_2_-induced gap junction remodeling.** Typical examples and quantitative results Cx43 immunofluorescence staining (n = 5). **P* < 0.05 vs. control group. #*P* < 0.05 vs. AF group. Cx43, connexin 43.


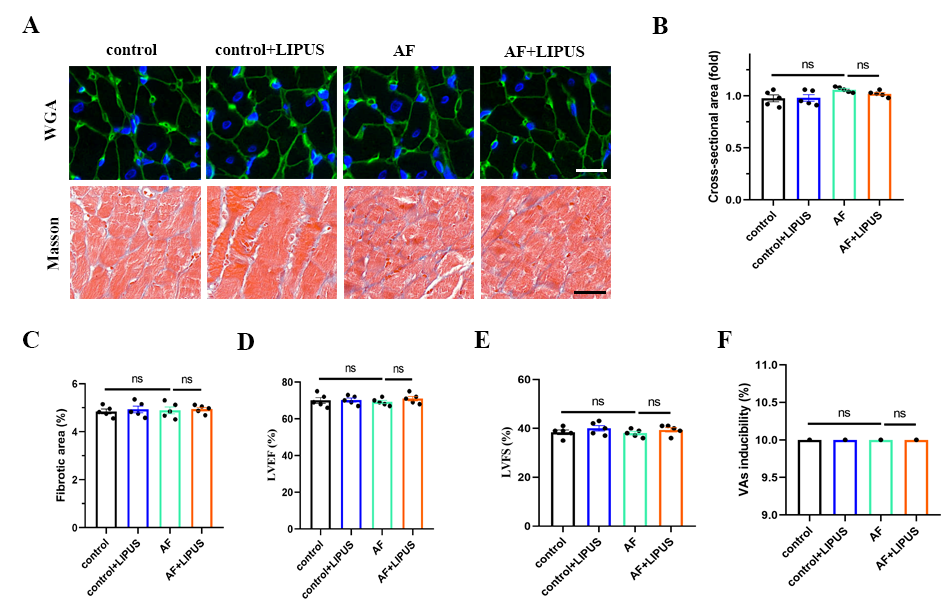


**Supplementary Figure S8. The effect of LIPUS on ACh/CaCl_2_-induced ventricular remodeling. (A-C)** Representative images of WGA and Masson-stained heart sections and quantitative of percentage of the fibrotic area (n = 5 per group). **(D-E)** Quantification of LVEF and LVFS (n = 5 per group). **(F)** Statistical analysis of VAs induced by burst-stimulating (n = 10 per group). **P* < 0.05.


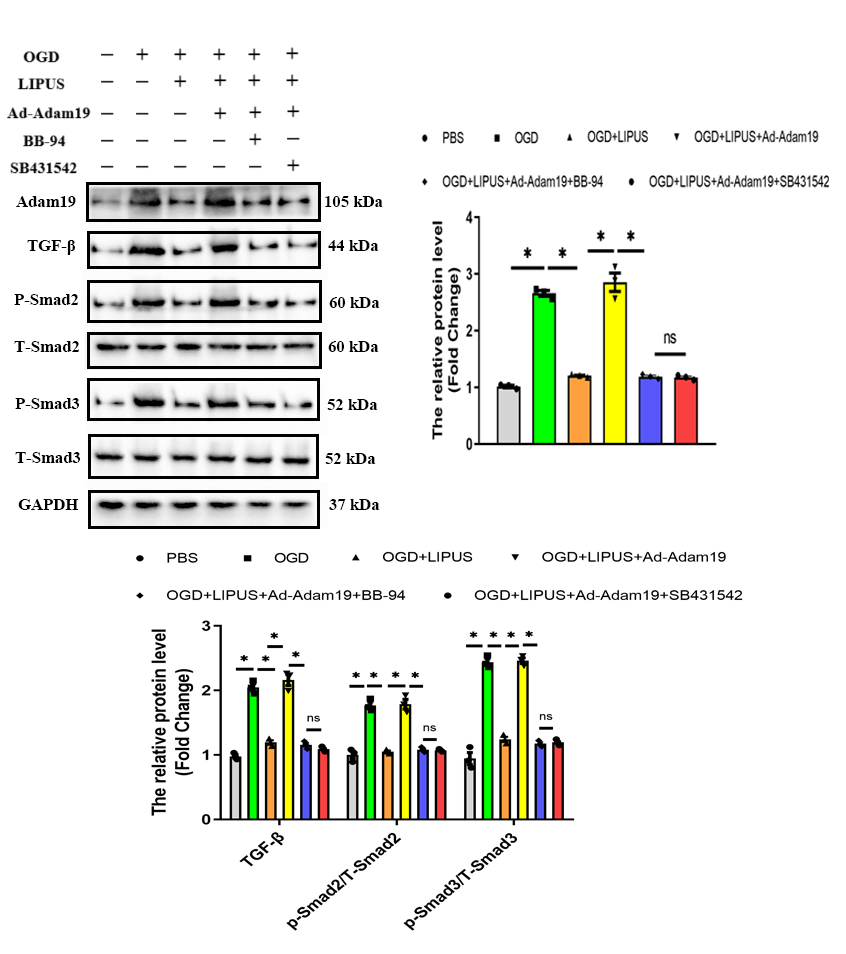
 **Supplementary Figure S9. The effect of LIPUS treatment on Adam19/ TGF-β/Smad2/3 signaling pathway *in vitro*.** Representative western blot and statistical results of Adam19, TGF-β, p-Smad2, T-Smad2, p-Smad3 and T-Smad3 protein expression (n = 3 per group). **P* < 0.05.


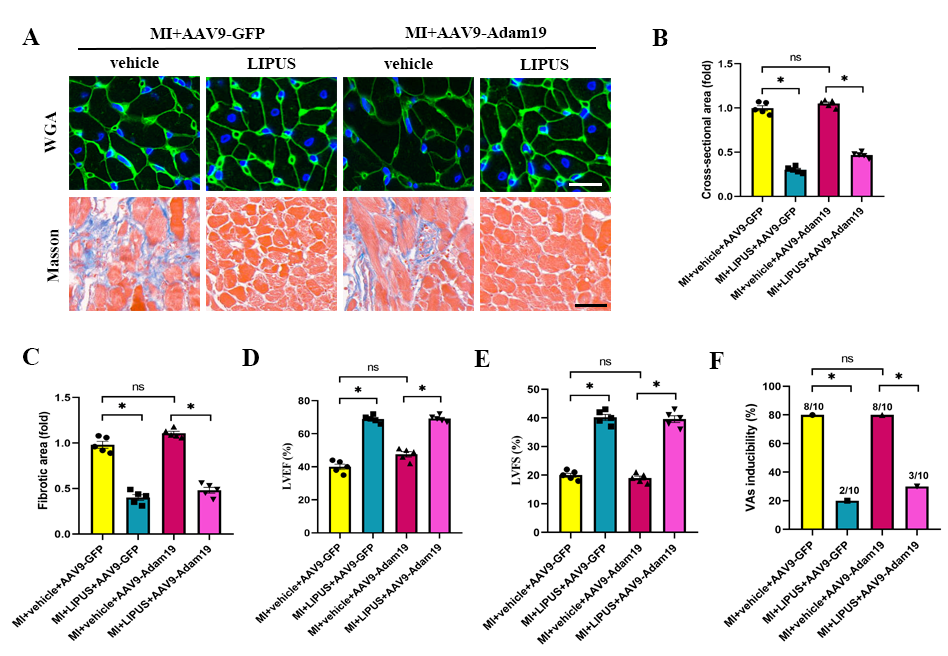


**Supplementary Figure S10. The effect of Adam19 overexpression on MI-induced ventricular remodeling. (A-C)** Representative images of WGA and Masson-stained heart sections and quantitative of percentage of the fibrotic area (n = 5 per group). **(D-E)** Quantification of LVEF and LVFS (n = 5 per group). **(F)** Statistical analysis of VAs induced by burst-stimulating (n = 10 per group). **P* < 0.05.


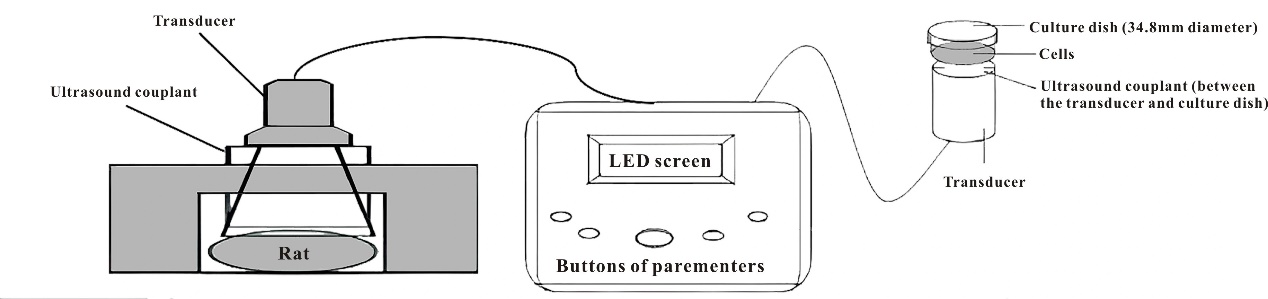


**Supplementary Figure S11.** Schematic diagram of LIPUS for treatments.

**3. Supplementary table**

**Supplementary Table S1**. Rat Primers for RT-PCR.

| Gene | Forward Primers | Reverse Primers |
| --- | --- | --- |
| Collagen-I | AAGACATCCCTGAAGTCAGC | CCTATGACTTCTGCGTCTGG |
| Collagen-III | ACAGCAGTCCAATGTAGATG | GAGCAGGTGTAGAAGGCTG |
| TGF-β | AATACGTCAGACATTCGGGAAGC | TCAATGTACAGCTGCCGTACAC |
| IL-1β | AGGAGAGACAAGCAACGACA | CTTTTCCATCTTCTTCTTTGGGTAT |
| IL-6 | AGTTGCCTTCTTGGGACTGATGT | GGTCTGTTGTGGGTGGTATCCTC |
| TNF-α | GCGTGTTCATCCGTTCTCTACC | TACTTCAGCGTCTCGTGTGTTTCT |
| GAPDH | GGACATTGTTGCCATCAACG | CTCCATGGTGGTGAAGACGC |

**Table S2**. Mouse Primers for RT-PCR.

| Gene | Forward Primers | Reverse Primers | |
| --- | --- | --- | --- |
| Collagen-I | CCG​TGA​CCT​CAA​GAT​GTG​CC | | GAA​CCT​TCG​CTT​CCA​TAC​TCG |
| Collagen-III | GAC​CTC​CTG​GAA​AAG​ATG​GAT​C | | AAA​TCC​ATT​GGA​TCA​TCC​CC |
| TGF-β | GTG​GCT​GAA​CCA​AGG​AGA​CG | | AGG​TGT​TGA​GCC​CTT​TCC​AG |
| IL-1β | TTCAGGCAGGCAGTATCACTC | | GAAGGTCCACGGGAAAGACAC |
| IL-6 | TGGAGTCACAGAAGGAGTGGCTAAG | | GACCACAGTGAGGAATGTCCAC |
| TNF-α | GAGAAAGTCAACCTCCTCTCTG | | GAAGACTCCTCCCAGGTATATG |
| GAPDH | CGC​TAA​CAT​CAA​ATG​GGG​TG | | TTG​CTG​ACA​ATC​TTG​AGG​GAG |

**Reference**

[1] Wang D, Wang W, Liang Q, et al. DHEA-induced ovarian hyperfibrosis is mediated by TGF-β signaling pathway. J Ovarian Res. 2018;11(1):6.

[2] Liu Y, Yin Z, Xu X, et al. Crosstalk between the activated Slit2-Robo1 pathway and TGF-β1 signalling promotes cardiac fibrosis. ESC Heart Fail. 2021;8(1):447-460.

[3] Badreldin H, Elshal M, El-Karef A, Ibrahim T. Empagliflozin protects the heart from atrial fibrillation in rats through inhibiting the NF-κB/HIF-1α regulatory axis and atrial remodeling. Int Immunopharmacol. 2024;143(Pt 2):113403.

[4] Su Y, Huang J, Sun S, et al. Restoring the Autonomic Balance in an Atrial Fibrillation Rat Model by Electroacupuncture at the Neiguan Point. Neuromodulation. 2024;27(7):1196-1207.

[5] Jiang X, Savchenko O, Li Y, et al. A Review of Low-Intensity Pulsed Ultrasound for Therapeutic Applications. IEEE Trans Biomed Eng. 2019;66(10):2704-2718.

[6] Liu DD, Ullah M, Concepcion W, Dahl JJ, Thakor AS. The role of ultrasound in enhancing mesenchymal stromal cell-based therapies. Stem Cells Transl Med. 2020;9(8):850-866.

[7] Qin H, Luo Z, Sun Y, et al. Low-intensity pulsed ultrasound promotes skeletal muscle regeneration via modulating the inflammatory immune microenvironment. Int J Biol Sci. 2023;19(4):1123-1145.

[8] Chen R, Du W, Zhang X, et al.Protective effects of low-intensity pulsed ultrasound (LIPUS) against cerebral ischemic stroke in mice by promoting brain vascular remodeling via the inhibition of ROCK1/p-MLC2 signaling pathway. Cereb Cortex. 2023;33(22):10984-10996.

[9] Guan M, Han X, Liao B, et al. LIPUS Promotes Calcium Oscillation and Enhances Calcium Dependent Autophagy of Chondrocytes to Alleviate Osteoarthritis. Adv Sci (Weinh). 2025;12(16):e2413930.

[10] Zhou D, Huang Z, Zhu X, Hong T, Zhao Y. Combination of endothelial progenitor cells and BB-94 significantly alleviates brain damage in a mouse model of diabetic ischemic stroke. Exp Ther Med. 2021;22(1):789.

[11] Cao J, Jiao M, Kou Z, Han F, Dong L. SB431542 partially inhibits high glucose-induced EMT by restoring mitochondrial homeostasis in RPE cells. Cell Commun Signal. 2024;22(1):17.

[12] Hu Y, Jia Y, Wang H, et al. Low-intensity pulsed ultrasound promotes cell viability and inhibits apoptosis of H9C2 cardiomyocytes in 3D bioprinting scaffolds via PI3K-Akt and ERK1/2 pathways. J Biomater Appl. 2022;37(3):402-414.

[13] Liu X, Qu C, Yang H, et al. Chronic stimulation of the sigma-1 receptor ameliorates autonomic nerve dysfunction and atrial fibrillation susceptibility in a rat model of depression. Am J Physiol Heart Circ Physiol. 2018;315(6):H1521-H1531.

[14] Yang HJ, Kong B, Shuai W, et al. Shensong Yangxin attenuates metabolic syndrome-induced atrial fibrillation via inhibition of ferroportin-mediated intracellular iron overload. Phytomedicine. 2022;101:154086.
